# Integration of the *Salmonella* Typhimurium Methylome and Transcriptome Reveals That DNA Methylation and Transcriptional Regulation Are Largely Decoupled under Virulence-Related Conditions

**DOI:** 10.1128/mbio.03464-21

**Published:** 2022-06-06

**Authors:** Jeffrey S. Bourgeois, Caroline E. Anderson, Liuyang Wang, Jennifer L. Modliszewski, Wei Chen, Benjamin H. Schott, Nicolas Devos, Dennis C. Ko

**Affiliations:** a Department of Molecular Genetics and Microbiology, School of Medicine, Duke University grid.471396.egrid.26009.3d , Durham, North Carolina, USA; b University Program in Genetics and Genomics, Duke University grid.471396.egrid.26009.3d , Durham, North Carolina, USA; c Center for Genomics and Computational Biology, Duke University grid.471396.egrid.26009.3d , Durham, North Carolina, USA; d Division of Infectious Diseases, Department of Medicine, School of Medicine, Duke University grid.471396.egrid.26009.3d , Durham, North Carolina, USA; University of Liverpool; University of Würzburg

**Keywords:** DNA methylation, *Salmonella*, gene regulation, m^6^A, methylome, transcription

## Abstract

Despite being in a golden age of bacterial epigenomics, little work has systematically examined the plasticity and functional impacts of the bacterial DNA methylome. Here, we leveraged single-molecule, real-time sequencing (SMRT-seq) to examine the m^6^A DNA methylome of two Salmonella enterica serovar Typhimurium strains: 14028s and a Δ*metJ* mutant with derepressed methionine metabolism, grown in Luria broth or medium that simulates the intracellular environment. We found that the methylome is remarkably static: >95% of adenosine bases retain their methylation status across conditions. Integration of methylation with transcriptomic data revealed limited correlation between changes in methylation and gene expression. Further, examination of the transcriptome in Δ*yhdJ* bacteria lacking the m^6^A methylase with the most dynamic methylation pattern in our data set revealed little evidence of YhdJ-mediated gene regulation. Curiously, despite G(m^6^A)TC motifs being particularly resistant to change across conditions, incorporating *dam* mutants into our analyses revealed two examples where changes in methylation and transcription may be linked across conditions. This includes the novel finding that the Δ*metJ* motility defect may be partially driven by hypermethylation of the chemotaxis gene *tsr*. Together, these data redefine the *S.* Typhimurium epigenome as a highly stable system that has rare but important roles in transcriptional regulation. Incorporating these lessons into future studies will be critical as we progress through the epigenomic era.

## INTRODUCTION

Until recently, systematically understanding how the bacterial DNA methylome affects physiology has been an unachievable task. Unlike eukaryotes where m**^5^**C DNA methylation is highly abundant and can be detected with bisulfate sequencing (1), bacterial genomes primarily house m^6^A methylation, which has historically been difficult to detect. Despite this technological hurdle, many studies over the last several decades have successfully uncovered roles for DNA methylation in contexts of restriction-modification systems (reviewed in reference 2), as well as for “orphan” methylases (particularly the Dam methylase) in DNA repair (3–9), DNA/bacterial replication and viability (10–19), *agn43* phase variation (20), LPS modifications (21–25), phage defense (21, 26, 27), mating (28, 29), fimbria formation (30, 31), antibiotic resistance (32), hypoxia survival (33), motility (17, 23, 31, 34), and other virulence-related processes (8–10, 16–19, 23, 31, 34–39). While orphan methylases were originally thought to regulate bacterial physiology, whereas restriction-modification systems targeted foreign DNA, recent work on “phasevarions” has shown that restriction-modification systems can indeed have a dramatic impact on the genome (reviewed in reference 40). A more complete history of associations between methylases and phenotypes can be found in recent reviews (41–43).

While early epigenome studies are valuable for the insights they provide, they depended on low-throughput and relatively blunt approaches (e.g., restriction enzyme digests paired with Southern blotting to infer methylation). These approaches could not be leveraged to address whether and how genome-wide changes in DNA methylation associate with changes to cellular processes. However, the discovery that sequencing data from Pac-Bio single-molecule, real-time sequencing (SMRT-seq) (44) and Oxford Nanopore sequencing (45, 46) systems can be repurposed to detect m^6^A has heralded a golden age of bacterial DNA methylomics. These technological breakthroughs were rapidly applied to cataloging bacterial methylomes, many of which have been deposited in publicly available databases such as REBASE (47). However, we have only recently seen the power of these third-generation sequencing technologies applied to connect DNA methylation to cellular phenotypes. For instance, a recent paper utilized SMRT-seq to identify specific changes in G(m^6^A)TC patterns within the *opvAB* promoter that are highly present in the population following phage insult (48), building on previous phenotypic observations (21). Other groups have leveraged comparative epigenomics to examine methylation patterns across isolates and identify potentially important trends in methylation (35, 49, 50). Thus, there is immense potential for SMRT-seq to identify how methylation correlates with impactful biology.

Despite these advances, we note that few studies have leveraged SMRT-seq to understand how methylation itself changes under different environmental pressures. Instead, the studies listed above typically examine methylomes under a single condition (typically late stationary-phase growth) to infer where methylation *can* happen, with notable exception (11, 32, 51–53). While informative, these approaches may not represent the methylation status of bacteria at growth phases typically studied in bacteriology and thus may have limited ability to integrate into the broader microbiological literature and with transcriptomic data sets. A related shortcoming of many methylation studies is that methylation sites in promoters are often reported as evidence of methylation-mediated regulation, without determining whether methylation is dynamic under relevant environmental changes or testing whether disrupting methylation at those sites impacts transcription. Curiously, while a number of classical approaches have identified examples of methylation at specific sites regulating gene expression (e.g., *pap* [30, 54, 55], *opvAB* [21], *agn43* [56, 57], *gtr* [22], the *std* operons [58], *dnaA* [12], *traJ* [29], and *sci1* [36]), we are unaware of any methylation site originally identified using genome-wide approaches that has been confirmed to impact gene expression. This disconnect between the technological advances in methylomics and the relatively modest conceptual advances in the field make it clear that the use of third-generation sequencing technologies to interrogate the DNA methylome is still in its infancy and that the important question of the generalizability of epigenetic regulation of transcription in bacteria should be addressed.

Here, we perform a series of SMRT-seq and transcriptome sequencing (RNA-seq) experiments to understand the role of DNA methylation in regulating Salmonella enterica serovar Typhimurium (*S.* Typhimurium) gene expression under environmental conditions critical for Salmonella virulence. Specifically, we studied conditions that activate the motility and Salmonella Pathogenicity Island-1 (SPI-1) pathways (growth in Luria broth [LB] until late log phase) and conditions that activate the Salmonella Pathogenicity Island-2 (SPI-2) pathways (growth in low phosphate and magnesium [LPM] medium [59] until late log phase). Since methionine metabolism is intimately connected to methylation, we also examined the changes in methylation associated with derepressed methionine metabolism using a Δ*metJ* mutant. In general, we found that DNA methylation is mostly stable across conditions and is broadly decoupled from gene expression changes. This study redefines our understanding of the *S.* Typhimurium epigenome, provides multiple epigenomic data sets that can be incorporated into future work to identify rare instances where methylation changes are coupled with transcription, and presents a basic blueprint for carrying out future methylomic studies with high reproducibility.

## RESULTS

### A genome-wide screen to understand how growth conditions and methionine metabolism impact m^6^A DNA methylation.

**FIG 1:**
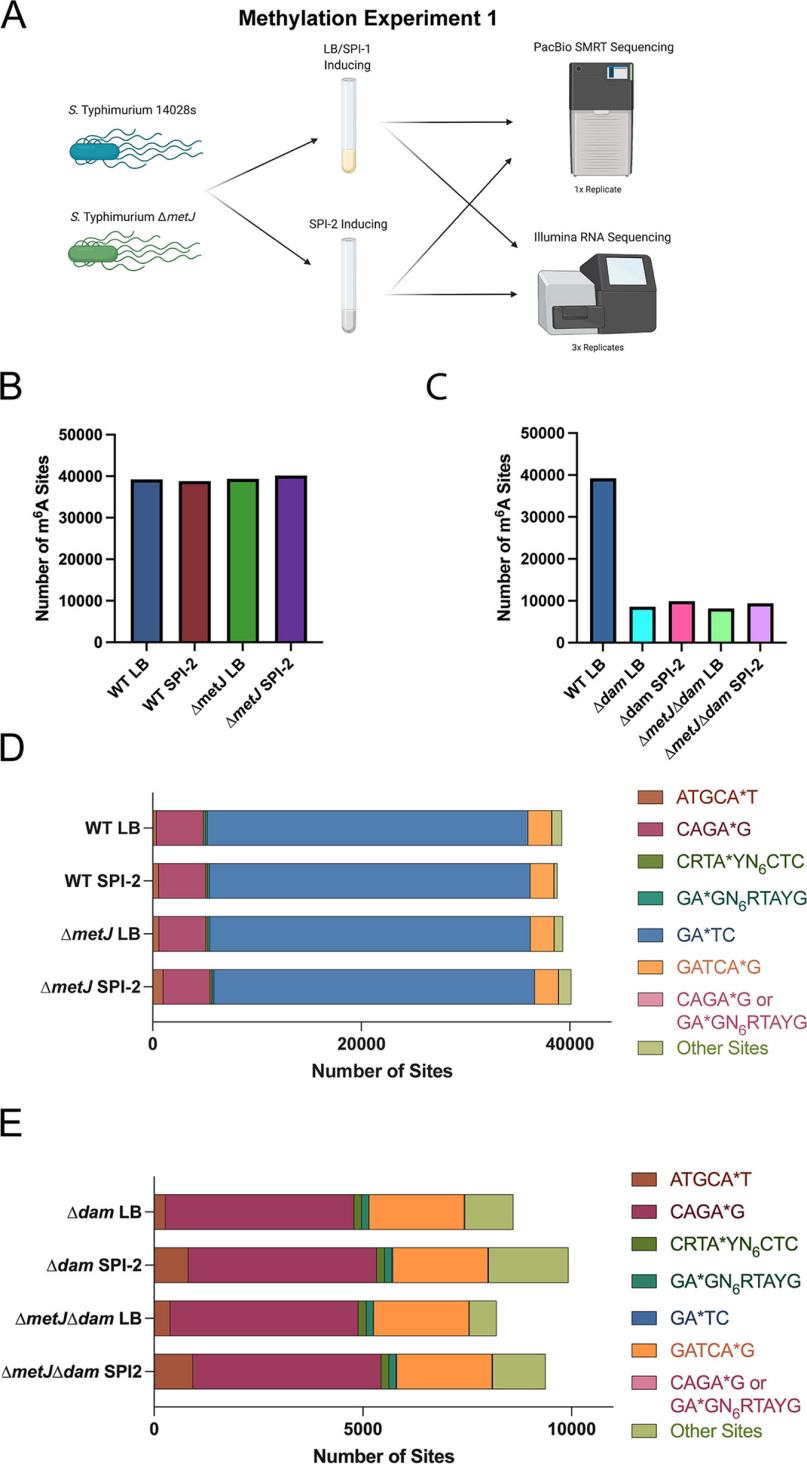
Genome-wide analysis of m^6^A DNA methylation under various conditions. (A) Schematic of methylation experiment 1. Wild-type *S.* Typhimurium (strain 14028s) and an isogenic Δ*metJ* strain were cultured in LB or SPI-2-inducing media, and DNA was collected for SMRT-seq. Bacteria grown under identical conditions were harvested for RNA-sequencing. (B and C) The total number of m^6^A bases observed across conditions does not dramatically change in wild-type and Δ*metJ* bacteria (B) but does change dramatically in Δ*dam* bacteria (C). (D) Analysis of motifs methylated reveals only the total number of ATGCA*T and “other” sites (sites that do not map to one of the six motifs) changes dramatically across conditions. The roughly 20 sites that could not be distinguished between CAGA*G or GA*GN_6_RTAYG methylation are listed as “CAGA*G or GA*GN_6_RTAYG.” (E) Δ*dam* mutation results in ablation of GATC methylation. For panels B through E, bases were only included in the analysis if the base could confidently be called methylated or unmethylated across the eight conditions.

While previous work on bacterial DNA methylation has largely focused either on global DNA methylation patterns under a single condition or on how methylation of a single motif changes under different conditions, we sought to examine how the entire *S.* Typhimurium m^6^A DNA methylome changes under four biologically relevant conditions (Fig. 1A). We examined aerobic growth in LB to late exponential phase (optical density at 600 nm [OD_600_] ~ 1.5 to 2.0), which induces expression of flagellar genes and the genes in the Salmonella Pathogenicity Island-1 (SPI-1)—including the type III secretion system used during host cell invasion (60). The second condition cultured bacteria in a minimal media used to induce expression of genes in the Salmonella Pathogenicity Island-2 (SPI-2) (59)—which include the type III secretion system turned on in the host cell to promote Salmonella vacuolar survival (61, 62). The third and fourth conditions repeated growth in these media but used a methionine metabolism mutant *S.* Typhimurium strain (Δ*metJ*). The MetJ protein represses expression of methionine metabolism genes (63). Thus, Δ*metJ* bacteria have deregulated methionine metabolism and accumulation of methionine and related metabolites, including metabolites directly related to methylation processes such as the universal methyl-donor *S*-adenosylmethionine (SAM) (64), and the methyltransferase-inhibiting metabolites methylthioadenosine (65–67) and *S*-adenosylhomocysteine (68–72). Of note, we have not only previously confirmed increased abundances of both SAM and methylthioadenosine in the Δ*metJ* mutant but also demonstrated that the Δ*metJ* mutant has attenuated SPI-1 secretion, motility, and virulence (73), and we had previously hypothesized that these effects could be mediated through aberrant methylation.

To analyze the DNA methylome, we performed a PacBio SMRT-seq experiment (here called “methylation experiment 1”) in a biological singlet (as has been common in the field and as we comment on below) to identify whether any changes in methylation could be observed. In this experiment, we also included Δ*dam* and Δ*dam* Δ*metJ* mutants, which lack G(m^6^A)TC (henceforth an asterisk [*] will denote the adenosine that is m^6^A modified, e.g., “GA*TC”) methylation grown under SPI-1 and SPI-2 conditions. This allowed us to confirm that our pipeline could adequately detect changes in methylation. These eight conditions were split across two PacBio SMRT cells. Thus, these conditions enabled comparison of the *S.* Typhimurium DNA methylome under the two conditions most critical for Salmonella virulence (SPI-1 and SPI-2 induction), following perturbation of methionine metabolism, and with a control condition ablating the primary DNA methyltransferase.

In total, this experiment defined the methylation status of 61,704 adenosine bases (GEO GSE185578); however, methylation status of some bases under certain conditions could not be determined since coverage was below 50×. Thus, we restricted our analysis to 51,177 bases in which the methylation status could be adequately determined for all conditions tested. These bases span both the *S.* Typhimurium genome and virulence plasmid.

To compare methylation across conditions, we called methylation in two ways. First, we assigned each base a “percent methylated” value, which considered the percentage of reads for each base that were counted as methylated compared to the total number of reads (see Data Set S1). We also examined the data as a binary value in which we considered bases either methylated (if any methylation was detected) or unmethylated (see Data Set S2). Using this binary analysis, we observed that there were similar, but subtly different, amounts of m^6^A methylation across wild-type (WT) and Δ*metJ* bacteria grown under LB and SPI-2 conditions (WT LB, 39,240 bases; WT SPI-2, 38,827 bases; Δ*metJ* SPI-1, 39,352 bases; Δ*metJ* SPI-2, 40,145 bases) (Fig. 1B), but that, as expected, the Δ*dam* mutation reduced total methylation substantially (Fig. 1C).

10.1128/mbio.03464-21.6 DATA SET S1 Quantitative methylation analysis. Download Data Set S1, XLSX file, 16.2 MB. Copyright © 2022 Bourgeois et al. 2022 Bourgeois et al. https://creativecommons.org/licenses/by/4.0/ This content is distributed under the terms of the Creative Commons Attribution 4.0 International license.

10.1128/mbio.03464-21.7 DATA SET S2 Binary methylation analysis. Download Data Set S2, XLSX file, 16.5 MB. Copyright © 2022 Bourgeois et al. 2022 Bourgeois et al. https://creativecommons.org/licenses/by/4.0/ This content is distributed under the terms of the Creative Commons Attribution 4.0 International license.

### ATGCAT motifs are frequently differentially methylated across conditions.

We next examined how these bases were distributed across different methylation motifs. This analysis detected methylation at motifs that had been previously detected in *S.* Typhimurium (74), though we were able to detect an additional motif, CRTA*YN_6_CTC, which appears to be the reverse complement of GA*GN_6_RTAYG. Notably, two motifs (CAGA*G and GA*GN_6_RTAYG) cannot always be distinguished, and so we included bases (~20 bases per condition) that matched both motifs in a separate category: “CAGA*G or GA*GN6RYAYG.” Bases that did not map to any known motif were listed as “Other.” As with the total amount of m^6^A methylation, we found that our four main conditions had subtle differences in the total numbers of most motifs (Fig. 1D).

The most notable change in motif abundance occurred at the ATGCA*T motif, which is methylated by the YhdJ methylase (75). We observed more ATGCA*T methylation in bacteria grown under SPI-2-inducing conditions (*P* < 0.00001, chi-square test) or in Δ*metJ* bacteria (*P* < 0.00001, chi-square test), with the highest ATGCA*T methylation present in Δ*metJ* bacteria grown under SPI-2-inducing conditions. We also observed variation in the number of bases that mapped to the “Other” category. In contrast, we observed very little change in the total amount of GA*TC methylation (methylated by Dam) across these physiologically relevant conditions, though the deletion of *dam* resulted in almost complete ablation of methylation at the GATC motif (Fig. 1E).

**FIG 2:**
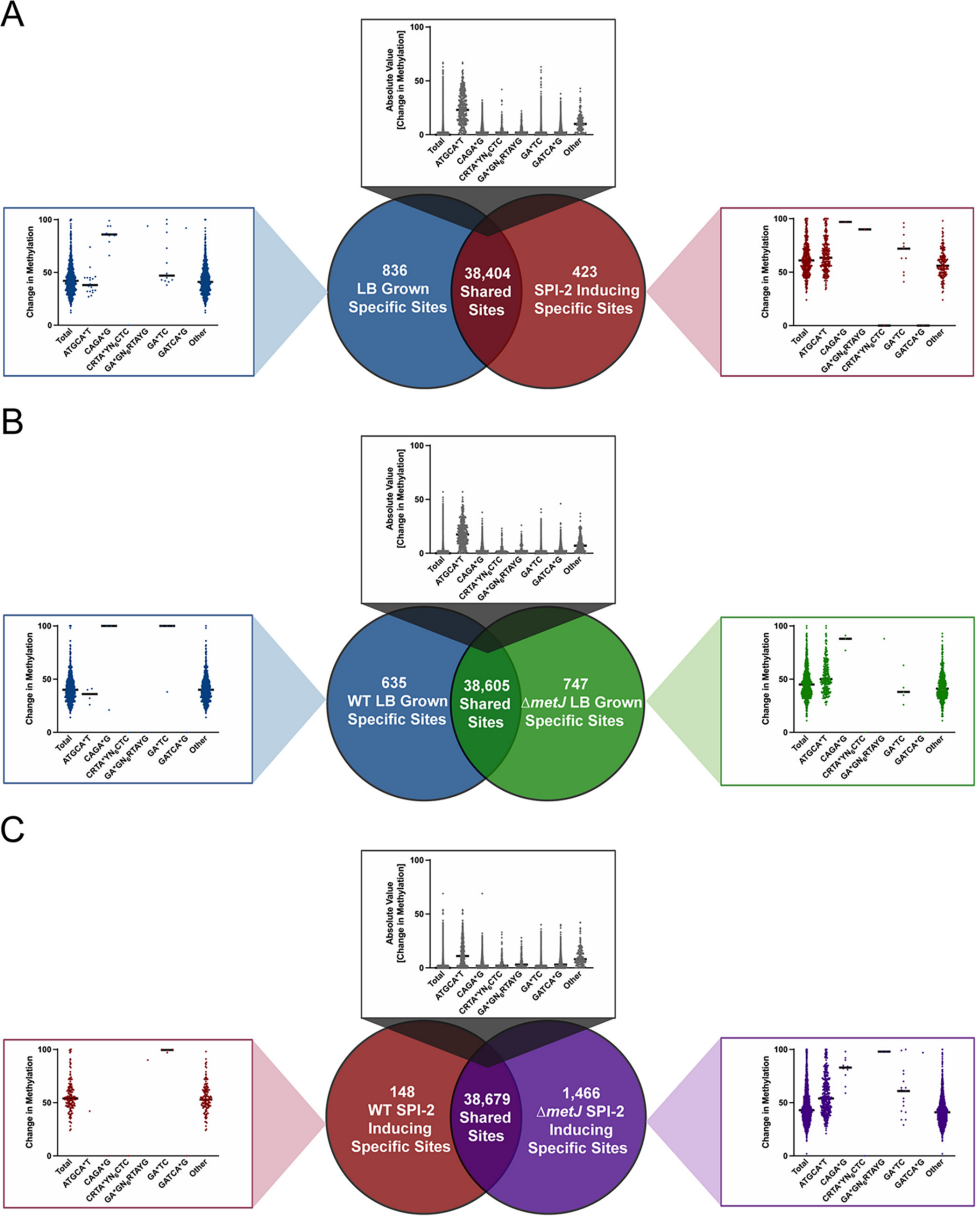
Integration of binary and quantitative analyses to understand differential methylation in *S.* Typhimurium. (A to C) Quantification of shared and unique methylated sites between wild-type *S.* Typhimurium grown in LB and SPI-2-inducing media (A), WT and Δ*metJ* bacteria grown in LB (B), and WT and Δ*metJ* bacteria grown in SPI-2-inducing media (C). Venn diagrams are based on binary measures of differential methylation. Sites identified by the binary analysis were examined in our quantitative data set in order to identify changes in the percent methylation. In the graphs, “Total” refers to all sites present in the relevant part of the Venn diagram, which were then broken down by motif. For motifs where no differentially methylated sites were present, a single dot is listed at 0%. For shared sites, the absolute value of the difference between bases are shown, and thus the numbers are agnostic to whether methylation is higher in either condition. Bars mark the median. For all panels, only bases that could be confidently called methylated or unmethylated under the eight conditions in Fig. 1 were considered.

To examine the methylation changes under LB versus SPI-2 inducing conditions with wild-type and Δ*metJ S.* Typhimurium, we compared binary methylation at each individual base to identify differentially methylated bases (bases that were called methylated in one condition but not another). While each condition had a few hundred to over a thousand bases that were not methylated in their opposing group, the vast majority of bases in this study (>38,000) were shared across these comparisons (Fig. 2A to C; Venn diagrams). This demonstrates that while the methylome is slightly responsive to the environment and methionine metabolism, it remains largely static across strikingly different conditions.

Having identified these differentially methylated bases by our binary analyses, we integrated our quantitative data. This is an important measurement as previous work has speculated that methylation impacts bistable gene expression (48, 76), and thus changes in the percentage of the population in which a given base is methylated could have implications on the percentage of the population expressing a given gene. For each differentially methylated base, we asked what the total change in methylation was across the two conditions (Fig. 2; see graphs in panels A to C). The total median shift in the percent methylation varied by condition, but fell between 43 and 53%, suggesting that most bases go from unmethylated in one condition, to about half the copies of the genome having methylation at that site in the other. Notably, most of the “shared” bases that demonstrated methylation under both conditions demonstrated no quantitative change (median = 0%). However, again the exception was ATGCA*T, where the median shift among shared bases remained relatively high (11 to 23%, depending on the condition).

To test for enrichment of motifs among differentially methylated bases, we compared the frequency of each of the six motifs tested above in the differentially methylated sites against the frequency observed in the entire condition. This analysis revealed that among the uniquely SPI-2-induced methylated bases, we observed 37 times more differentially methylated ATGCA*T sites than expected. Similarly, sites methylated in Δ*metJ S.* Typhimurium, but not wild-type bacteria, grown under both LB and SPI-2-inducing conditions are also dramatically enriched for YhdJ-mediated methylation (20- and 11-fold enrichment, accordingly) (see Fig. S1A in the supplemental material). Surprisingly, all other motifs were either present at similar or dramatically lower abundance among differentially methylated sites than expected by chance, though we did note significant enrichment of “other” motifs among differentially methylated bases (20- to 100-fold enrichment, depending on the condition).

10.1128/mbio.03464-21.1 FIG S1 Supplemental analyses and follow-up data. (A) Identification of motifs enriched in methylation sites unique to each of the comparisons in Fig. 2. Motif enrichment was calculated by dividing the frequency of the motif among the uniquely methylated bases by the genome-wide frequency of that motif within that condition, e.g., for panel A, the frequency of ATGCA*T within unique WT SPI-2 sites = 242 ATGCA*T sites/423 unique SPI-2 sites (0.57); the frequency of ATGCA*T within all WT SPI-2 = 600 ATGCA*T sites/38,843 detected motifs (0.015); (enrichment = 0.57/0.015 = 37.04). For all panels, only bases that could be confidently called methylated or unmethylated in all eight conditions in methylation experiment 1 were considered. (B and C) ACCWGG is enriched in “other” differentially methylated sites. The 40-bp flanking sites that were differentially methylated between wild-type and Δ*metJ* bacteria grown in LB in our combined dataset (B) or between wild-type bacteria grown in LB and SPI-2-inducing conditions in methylation experiment 1 (C) but did not map to one of our six motifs were plugged into the MEME software (77) in order to identify overrepresented motifs. ACCWGG, a common miscall for the m^5^C motif CCWGG, was identified in both comparisons. (D) PCA analysis comparing data from the Δ*metJ* RNA-seq experiment (see Data Set S4, Exp 1) and the Δ*yhdJ* experiment (see Data Set S5, Exp 2) cluster with data from Kröger et al. (78). The LB condition used from Kröger et al. was early stationary phase, for which the OD_600_ (~2.0) most closely matches the OD_600_ used in this study (1.5 to 2.0). Both the SPI-2-inducing condition and the SPI-2+MgCl_2_ condition were included from Kröger et al. Media separate along PC1, which accounts for 53.5% of the variation. Each condition from this paper is completed in triplicate (with three dots represented on the plot), each Kröger condition in duplicate (two dots on the plot). (E and F) *flhDC* expression is reduced in Δ*metJ*. (E) In contrast to our previous findings (73), *flhD* expression is reduced in Δ*metJ* bacteria by qPCR. Bacteria in late-log-phase growth in LB were harvested, RNA was stabilized, and RNA was extracted and quantified as described in the methods. Fold change (Δ*metJ*/wild type) is expressed as 2^–ΔΔ^*^CT^*, where the *flhD* transcript was normalized to the *rrs* gene. Each dot represents the average of two to three technical replicates. (F) Endogenous tagging of *flhC* confirms reduced *flhDC* expression in Δ*metJ* bacteria. A C-terminal 3×FLAG tag was added to the *flhC* gene, and the abundance of the protein was measured by Western blotting. The specificity of the FLAG antibody to FlhC-3×FLAG was confirmed by comparison to a wild-type, untagged *S.* Typhimurium strain. FlhC-3×FLAG abundance was quantified after correcting for loading by normalizing to total protein, and data are presented relative to wild-type (WT) *flhC-3×FLAG S.* Typhimurium. Each dot represents an independent experiment, bars represent the mean, and error bars the standard error of the mean. For panels E and F, *P* values are from a one-sample *t* test performed on the log-transformed data comparing the log values to 0. (G to J) Differentially methylated sites from Sánchez-Romero et al. (76) do not correlate with reproducible changes in gene expression. (G and H) *carA* (G) and *dgoR* (H) do not show reproducible changes in gene expression between LB grown and SPI-2-induced bacteria. (H and I) Other genes have reproducible effects in the dataset, including (H) *prgH* and (I) *ssaT.* “Exp 1” refers to the Δ*metJ* RNA-seq experiment (see Data Set S4), and “Exp 2” refers to the Δ*yhdJ* RNA-seq experiment (see Data Set S5). The *P* value was calculated from the FDR. (K) Methylation upstream of *flhDC* does not contribute to the Δ*metJ* motility defect. The −278 GATC sequence is not required for the impacts of Δ*metJ* on motility. Motility on soft agar was measured 6 h after inoculating the agar and after migration at 37°C, each dot represents the average of three to five technical replicates, data were normalized to the grand mean prior to plotting or performing statistics, and *P* values were generated by two-way ANOVA with Sidak’s multiple-comparison test. Download FIG S1, TIF file, 2.8 MB. Copyright © 2022 Bourgeois et al. 2022 Bourgeois et al. https://creativecommons.org/licenses/by/4.0/ This content is distributed under the terms of the Creative Commons Attribution 4.0 International license.

### A replication experiment demonstrates that SMRT-seq is highly reproducible and confirms differential methylation is predominantly driven by YhdJ.

**FIG 3:**
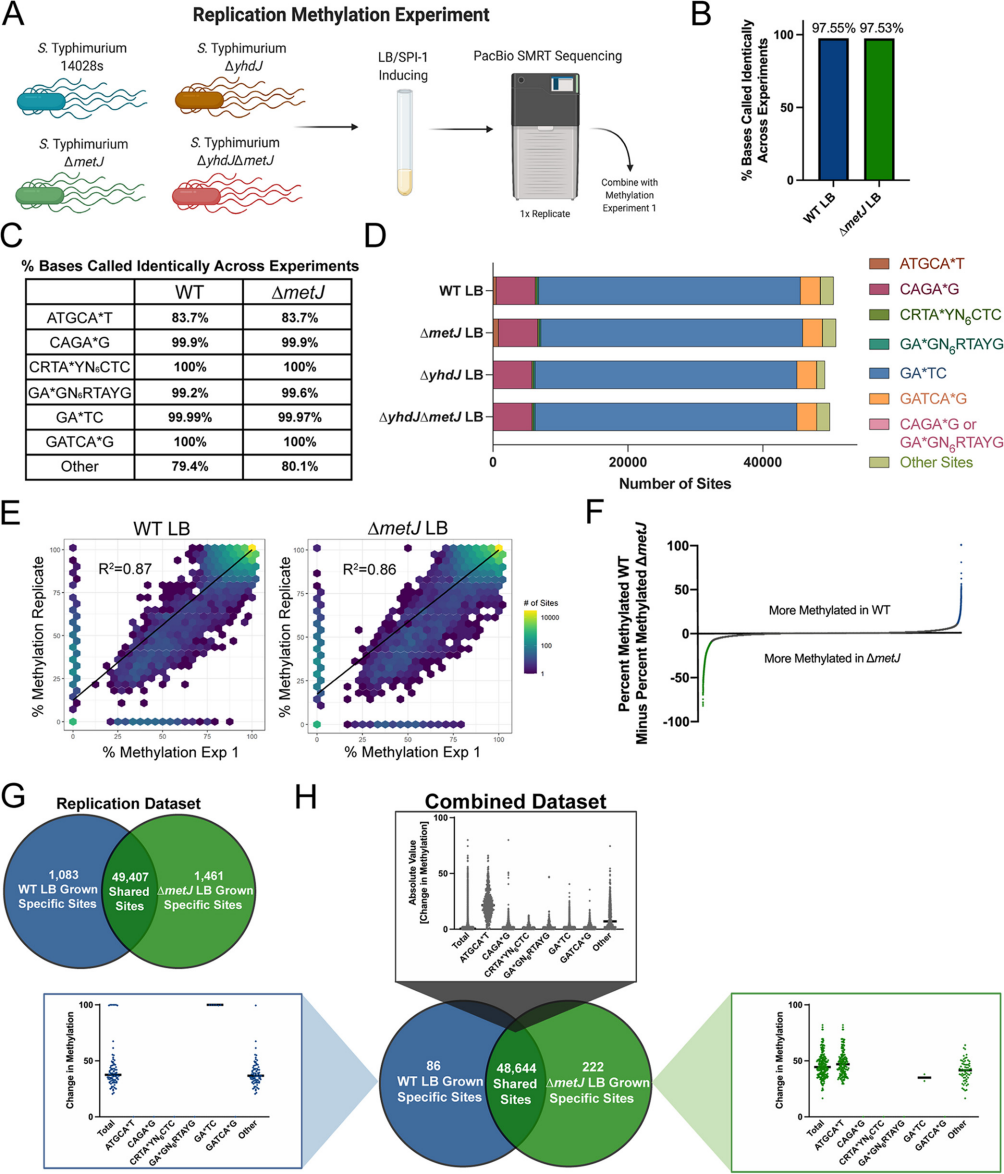
A replication screen reveals methylation is highly reproducible across SMRT-seq experiments but highlights the value of performing biological replicates. (A) Schematic for the replication methylation experiment. Wild-type *S.* Typhimurium (strain 14028s) or isogenic mutants were grown in LB media, and DNA was harvested for SMRT-seq. (B) Approximately 97% of all bases were called identically (methylated or unmethylated) in methylation experiment 1 and the replicate methylation experiments. (C) ATGCA*T and other sites had lower rates of replication compared to other motifs. (D) Only ATGCA*T and “other” sites (bases that do not map to one of the six motifs) change dramatically across tested conditions in the replication methylation experiment. No ATGCA*T methylation was observed in Δ*yhdJ* mutants. (E) The observed percent methylation at each base was reproducible across experiments. The color of the hexagon represents the number of bases that fall at that point on the axes. *R*^2^ values and trendlines represent the correlation across experiments. (F) Quantitative analysis reveals numerous sites are differentially methylated between wild-type and Δ*metJ* bacteria. Each dot represents the mean percent methylation in wild-type bacteria across the two experiments subtracted by the mean methylation in Δ*metJ* bacteria for each adenosine confidently called in both experiments. Blue and green dots mark bases where the mean difference is ≥10%. (G) Quantification of unique methylation sites in the replication experiment. For panels D to G, bases were only included in the analysis if the base could confidently be called methylated or unmethylated across conditions. (H) Venn diagram is based on binary measures of differential methylation in the combined data set. Sites identified by the binary analysis were examined in our quantitative data set in order to identify changes in the percent methylation. In the graphs, “Total” refers to all differentially methylated sites under that condition, and differentially methylated sites are then broken down by motif. For motifs where no differentially methylated sites were present, a single dot is listed at 0%. For shared sites, the absolute value of the difference between bases are shown and thus the numbers are agnostic to whether methylation is higher in either condition. Bars mark the median.

To confirm our findings that DNA methylation was largely stable among our conditions with the exception of ATGCA*T sites, we repeated our SMRT-seq experiment with wild-type and Δ*metJ* bacteria grown in LB (Fig. 3A, replication methylation experiment). Further, to confirm that the significant enrichment in ATGCA*T methylation in Δ*metJ* bacteria that we observed above was due to YhdJ, we also sequenced Δ*yhdJ* and Δ*yhdJ *Δ*metJ S.* Typhimurium grown in LB. Of note, although we sequenced eight samples across two SMRT cells in methylation experiment 1, we sequenced these four samples on two SMRT cells, significantly increasing our sequencing depth. The resulting data set called the methylation status of 60,502 bases under at least one condition; strikingly, 60,501 of these bases were confidently called in all four conditions (GEO GSE185501).

Analysis of the two methylomic data sets using our binary assessment revealed that ~97.5% of bases replicated their methylation status across experiments, demonstrating that our results were highly reproducible (Fig. 3B; see Data Set S2). Of note, sites assigned to ATGCA*T or “other” motifs may include significantly more miscalled methyl bases, as only ~80% (79.3% of wild-type strains and 80.1% of Δ*metJ* strains) “other” bases replicated their methylation status across experiments (Fig. 3C). Alternatively, as we see these bases are more dynamic across our conditions, these motifs may represent biologically meaningful sources of variation across experiments. Importantly, we again observed that Δ*metJ* bacteria have increased ATGCA*T methylation and found 0 methylated ATGCA*T sites in Δ*yhdJ* and Δ*yhdJ *Δ*metJ* mutants, confirming that YhdJ is the only ATGCA*T methylase active in both bacterial strains (Fig. 3D).

Analysis of the two data sets using the quantitative measurement (see Data Set S3) revealed considerable replication across the data sets (wild type, *R*^2^ = 0.87; Δ*metJ*, *R*^2^ = 0.86) (Fig. 3E). Considering these experiments as separate biological replicates and using an arbitrary cutoff of 10% average differential methylation, we identified 2,528 sites (of 50,962 total sites; 4.96%) that were differentially methylated between wild-type and Δ*metJ* bacteria using this quantitative method (Fig. 3F). A total of 881 of these sites were more methylated in wild-type bacteria, and 1,647 were more methylated in Δ*metJ* bacteria.

10.1128/mbio.03464-21.8 DATA SET S3 Combined quantitative analysis. Download Data Set S3, XLSX file, 6.8 MB. Copyright © 2022 Bourgeois et al. 2022 Bourgeois et al. https://creativecommons.org/licenses/by/4.0/ This content is distributed under the terms of the Creative Commons Attribution 4.0 International license.

Having assessed the reproducibility of SMRT-seq for both categorical and quantitative measures of methylation, we used our binary measurement to generate a combined data set containing bases which were (i) reliably detected in wild-type and Δ*metJ* bacteria grown in LB in both experiments and (ii) were identically called methylated or unmethylated in both experiments. Using this data set (52,594 bases), we determined which differentially methylated bases repeated across the two studies. This number of bases is greater than the number of bases included in methylation experiment 1 analyses, since we no longer needed to exclude bases that did not reach sufficient coverage under either the SPI-2-inducing or Δ*dam* conditions. While our data demonstrated that the vast majority of bases were called identically (~97.5%; Fig. 3B), we found that a disproportional number of bases that were called differentially methylated in the pilot study failed to replicate in the replication study, and vice versa. This is in line with our observation that there was reduced reproducibility in our more dynamic ATGCA*T and “other” motifs (Fig. 3C). In fact, while there were 1,382 bases called differentially methylated in the first experiment (Fig. 2B), and 2,544 bases called differentially methylated in the replicate study (Fig. 3G), only 308 differentially methylated bases were identified in the combined data set (Fig. 3H). Importantly, the overlap between these two replicates is much greater than expected by chance (3.7-fold enrichment; *P* < 0.0001, one-tailed binomial test), indicating that these biological replicates provide a high-confidence set of 308 differentially methylated sites, though some false positives likely remain. Our findings emphasize that while SMRT-seq calling of methylated bases is reliable, replication is especially important in examining bases that change between conditions.

Of note, the combined data set once again revealed enrichment of differentially methylated “other” sites. To understand these sites, we examined the 40 bases surrounding the 143 instances of “other” differential methylation in the combined data set using “Multiple Em for Motif Elicitation” (MEME) software (77). This identified a single significant motif (E value = 6.1 × 10^−16^), ACCWGG (see Fig. S1B). The same motif was identified among the 969 differentially methylated “other” sites between LB and SPI-2 grown bacteria (methylation experiment 1; E value = 5.5 × 10^−206^; see Fig. S1C). The ACCTGG motif has been reported on multiple Salmonella serovar entry pages on REBASE (47); however, curators note that this is almost certainly a miscall for the m^5^C motif CCWGG—methylated by Dcm. Across the combined data set, we found 33 instances of this motif (23% of all differentially methylated “other” sites). This leads us to hypothesize that this dynamic “other” category may be predominantly driven by changes in the flexible m^5^C methylome, which warrants further investigation using sequencing technologies better equipped to detect cytosine methylation.

### There is no association between the transcriptome and the genome-wide binary methylation analyses under the conditions tested here.

Canonically, changes in DNA methylation can lead to changes in transcription by enabling differential binding of transcription factors to genomic elements (reviewed in reference 41). However, studies that describe this in bacteria typically either (i) focus on single loci (see, for example, reference 48), or (ii) only speculate on direct mechanisms of transcriptional control based on methylase knockout experiments (see, for example, reference 35). No study has directly examined whether differential methylation across the *S.* Typhimurium genome correlates with differential expression under biologically relevant conditions. We attempted to fill this gap in knowledge by performing RNA-seq on wild-type and Δ*metJ* bacteria grown in LB and SPI-2-inducing media (GEO GSE185072; see Data Set S4) and looking for correlations with our SMRT-seq data sets.

10.1128/mbio.03464-21.9 DATA SET S4 Δ*metJ* RNA-seq. Download Data Set S4, XLSX file, 2.6 MB. Copyright © 2022 Bourgeois et al. 2022 Bourgeois et al. https://creativecommons.org/licenses/by/4.0/ This content is distributed under the terms of the Creative Commons Attribution 4.0 International license.

**FIG 4:**
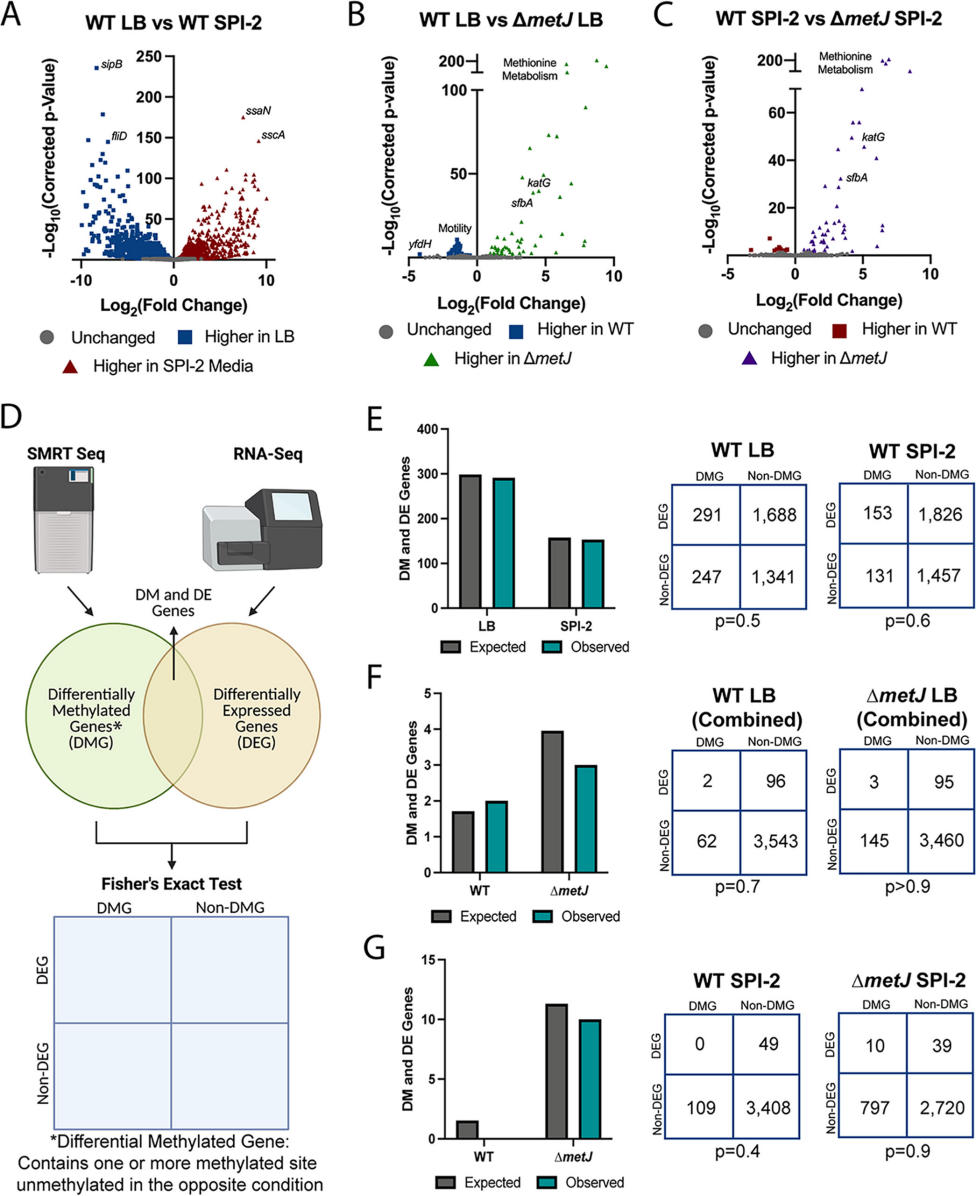
Differentially methylated genes by binary analysis are not enriched for transcriptomic changes (A to C) RNA-seq reveals transcriptomic changes between LB grown and SPI-2 media grown wild-type bacteria (A), wild-type and Δ*metJ* bacteria grown in LB (B), and wild-type and Δ*metJ* bacteria grown in SPI-2 media (C). Corrected *P* values generated by calculating the FDR. (D) Schematic of RNA-seq and SMRT-seq integration. Each gene was determined to be differentially methylated (differentially methylated gene [DMG]) in our binary analysis, differentially expressed (differentially expressed gene [DEG]; FDR < 0.05), differentially methylated and differentially expressed (DM and DE gene), or neither differentially methylated nor expressed. A Fisher exact test was then used to determine whether there was an association between methylation and gene expression. (E to G) Differential methylation is not associated with differential expression. Observed and expected numbers of differentially methylated and differentially expressed genes were not significantly different when comparing uniquely methylated genes in LB versus SPI-2 media (E), wild-type versus Δ*metJ* in LB (F), or wild-type versus *ΔmetJ* bacteria in SPI-2 media (G). Uniquely methylated genes are plotted in the condition under which they are methylated (e.g., for panel E, a gene that contains a base that is methylated in LB but not SPI-2 media would be plotted as part of “LB”) but are agnostic to the direction of effect for the expression data. Expected values are calculated by multiplying the frequency of differential methylation by the frequency of differential expression by the total number of genes in the analysis for each condition. Numbers used for the Fisher exact test are shown on the right. Data for panels E and G used data from methylation experiment 1, panel F used the “combined data set.” For panels F and G, the gene *metJ* is removed from the analysis, as it is artificially called both differentially methylated and expressed due to its excision from the genome.

Prior to integrating our data sets, we confirmed our RNA-seq data matched previously observed trends. As expected, we identified many differentially expressed genes (DEGs; 2,639 DEGs at a false discovery rate [FDR] of <0.5) between wild-type bacteria grown in LB versus SPI-2-inducing conditions (Fig. 4A). These DEGs included a variety of expected genes, including higher expression of SPI-1 genes (e.g., *sipB*, 322-fold induction) and flagellar genes (e.g., *fliD*, 138-fold induction) in LB and higher expression of SPI-2 genes (e.g., *ssaN* and *sscA;* 182- and 579-fold induction, respectively) in SPI-2-inducing media. Further, these data cluster with previous transcriptomic analyses of gene expression in LB and SPI-2-inducing conditions (78) by principal component analysis (PCA; 53.5% of variation between the two studies explained by media; see Fig. S1D). Analysis of Δ*metJ* DEGs revealed a number of expected trends, specifically that during growth in LB and SPI-2, Δ*metJ* bacteria show upregulation of methionine metabolism genes resulting from direct derepression of the metabolic pathway (Fig. 4B and C) (63). Of note, we also observed reduced motility gene expression which we had previously reported (73) but were surprised that, in contrast to our prior work, we observed a small reduction in *flhD* expression in the Δ*metJ* mutant. However, we confirmed this result by qPCR and Western blotting (see Fig. S1E and F) and speculate that improved DNase treatment in this study likely explains this difference.

To integrate our differential expression data with our methylomics data, we considered genes that either (i) contained or (ii) were the closest gene to one or more binary differentially methylated sites (i.e., present at any level in one condition, absent in the other) to be “differentially methylated genes” (DMGs) (Fig. 4D). For each comparison, the status “differentially methylated” applied to the condition in which the methyl mark was present (e.g., when comparing LB-grown versus SPI-2-grown bacteria, an LB-grown DMG contains a methyl mark that is absent in SPI-2-grown bacteria). Using these criteria, we examined whether differentially methylated genes were more likely to be differentially expressed than predicted by chance. Strikingly, we did not observe enrichment of DEGs among our DMGs. The number of DEGs that were also DMGs under all comparisons was remarkably similar to the overlap of these categories expected by chance (Fisher *P* value > 0.05 in all cases), suggesting the two phenomena are typically not associated at the genome-wide level across any of our comparisons with binary calling of DMGs (Fig. 4E to G). Most importantly, there was no evidence of enrichment comparing WT *S.* Typhimurium grown in LB (SPI-1 inducing) and SPI-2 conditions (Fig. 4E), indicating that cooccurrence of differential methylation and differential gene expression is not observed more frequently than is expected by chance in switching between these two critical growth conditions in Salmonella pathogenesis. Notably, we also did not observe correlations if we adjusted the statistical thresholds for differential expression (see Fig. S2A to D), or if we stratified our data by the differential expression direction of effect (see Fig. S2E and F), the genic location of the differential methylation (see Fig. S2G), or to specific motifs (see Fig. S2H and I).

10.1128/mbio.03464-21.2 FIG S2 Stratification of integrated data does not reveal additional associations between differentially expressed genes and differentially methylated genes. (A to I) Stratified binary data do not reveal additional associations. (A to C) The Fisher exact test does not reveal an association between differential expression and methylation when the statistical cutoff for differential expression is changed to a log_2_FC of >1.5 and an FDR-corrected *P* value of <0.05 for any condition. For wild-type LB versus SPI-2 comparisons, there are also no statistical associations when (D) the statistical cutoff for differential expression is changed to log_2_FC > 2.0, or when the data are stratified by (E) the direction of gene expression change in LB, (F) the direction of gene expression change in SPI-2 media, (G) differentially methylated bases upstream of genes, (H) differentially ATGCA*T methylation, or (I) differential GA*TC methylation. Uniquely methylated genes are plotted in the condition under which they are methylated (e.g., for panel B, a gene that has a methylated upstream base in LB but not SPI-2 media would be plotted as part of “LB”), but are agnostic to the direction of effect for the expression change except in panels E and F. Data for panel B from the combined dataset, all other data from methylation experiment 1. (J to R) Stratified quantitative data do not reveal additional associations. (J to L) The Fisher exact test does not reveal an association between differential expression and methylation when the statistical cutoff for differential expression is changed to a log_2_FC of >1.5 and an FDR-corrected *P* value of <0.05 for any condition, except for wild-type bacteria with increased methylation relative to Δ*metJ* bacteria in SPI-2 media where an association was also seen with the less stringent cutoff. (M to O) A Fisher exact test does not reveal an association between differential expression and methylation differentially methylated bases when only differential methylation upstream of genes is considered. (P-R) A Fisher exact test does not reveal an association between differential expression and methylation when the definition of “differential methylation” is shifted to sites where (i) the base is ≥99% methylated in one condition, and (ii) has a difference of ≥10% between the two conditions, except for wild-type bacteria with increased methylation relative to Δ*metJ* bacteria in SPI-2 media where an association was also seen with the less stringent cutoff. Differentially methylated genes are plotted in the condition under which they are hypermethylated (e.g., for panel M, a gene that has an upstream base with increased methylation in LB but not SPI-2 media would be plotted as part of “LB”), but are agnostic to the direction of effect for the expression change. Data for panels B, N, and Q from the combined dataset, all other data from methylation experiment 1. Download FIG S2, TIF file, 2.0 MB. Copyright © 2022 Bourgeois et al. 2022 Bourgeois et al. https://creativecommons.org/licenses/by/4.0/ This content is distributed under the terms of the Creative Commons Attribution 4.0 International license.

### There is limited association between the transcriptome and the genome-wide quantitative methylation analyses under the conditions tested here.

**FIG 5:**
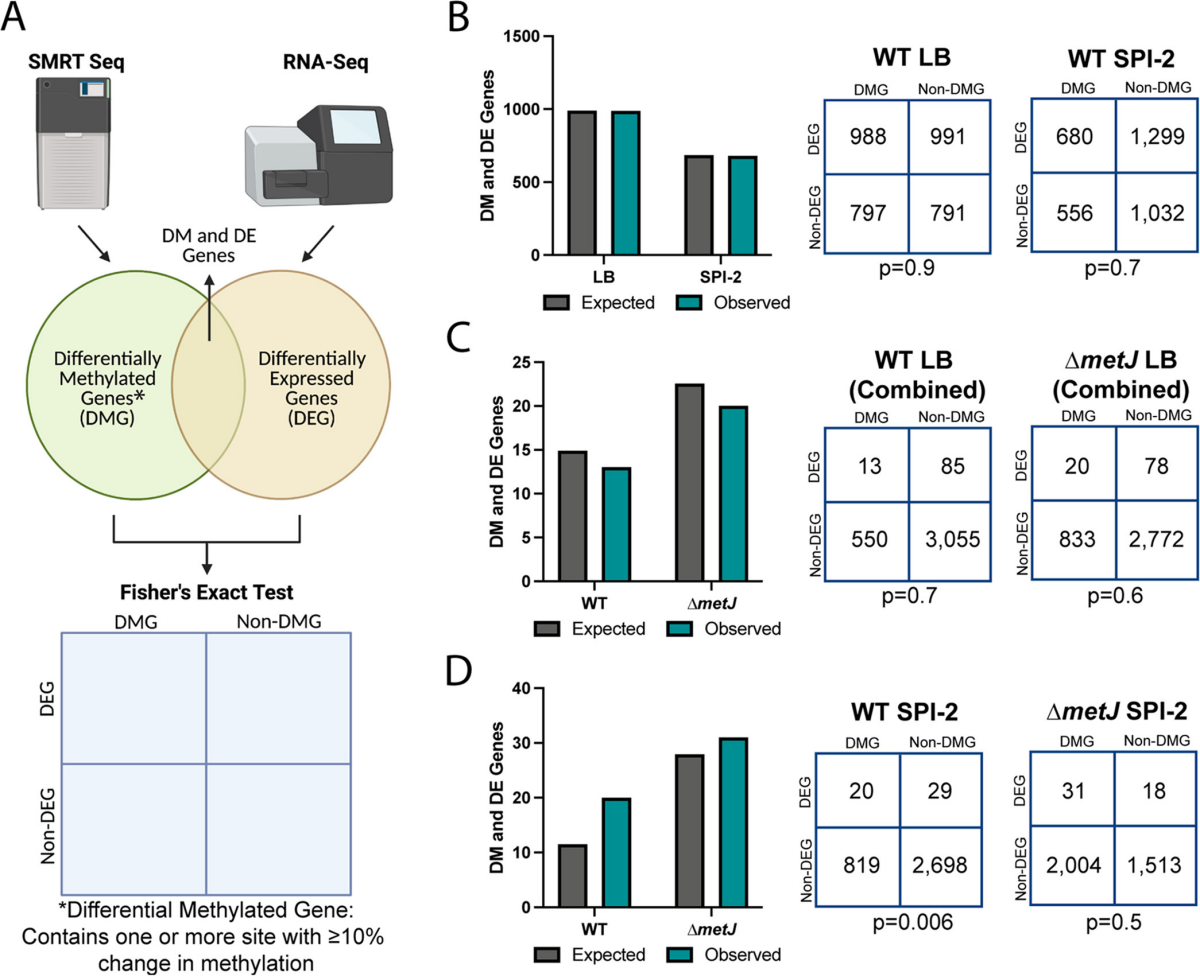
Quantitative analysis revealed an association between differential methylation and expression between wild-type and Δ*metJ* bacteria. (A) Schematic of RNA-seq and SMRT-seq integration. Each gene in our quantitative analysis was determined to be differentially methylated (differentially methylated gene [DMG]: difference ≥10% methylation across conditions), differentially expressed (differentially expressed gene [DEG]: FDR corrected *P* value ≤0.05), differentially methylated and differentially expressed (DM and DE Gene), or neither differentially methylated nor expressed. A Fisher exact test was then used to determine whether there was an association between methylation and gene expression. (B to D) Differential methylation is typically not associated with differential expression. Observed and expected numbers of differentially methylated and differentially expressed genes were not significantly different when comparing uniquely methylated genes in LB versus SPI-2 media (B) or wild-type versus Δ*metJ* bacteria in LB (C); however, a significant enrichment of DEGs was observed in hypermethylated sites in wild-type bacteria grown in SPI-2 media relative to *ΔmetJ* (D). Hypermethylated genes are plotted in a condition under which they have increased methylation (e.g., for panel B, a gene that contains a base that is more methylated in LB would be plotted as part of “LB”) but are agnostic to the direction of effect for the expression data. Expected values are calculated by multiplying the frequency of differential methylation by the frequency of differential expression by the total number of genes in the analysis for each condition. Numbers used for the Fisher exact test are shown on the right. Data for panels B and D used data from methylation experiment 1; data for panel C used the “combined data set.” For panels C and D, the gene *metJ* was removed from the analysis, as it is excised from the genome.

In addition to these binary definitions to differential methylation, we examined whether there was enrichment of DEGs among DMGs defined by a difference in ≥10% methylation across conditions (Fig. 5A). Most of our binary observations replicated in this analysis (Fig. 5B and C), but we noted an association between DEGs and DMGs in wild-type bacteria grown in SPI-2-inducing media compared to Δ*metJ* bacteria grown under the same conditions (Fig. 5D and Table 1). It is unclear why this condition is the exception to the general lack of association we observe, but it may suggest that methylases (particularly the *dam* methylase) and deregulated transcriptional machinery uniquely compete for access to these sites exclusively in minimal media. We also examined whether adjusting our thresholds for differential expression or limiting our search for differential to bases upstream of differentially expressed genes could reveal further correlations between expression and methylation, but no additional associations were found (see Fig. S2J to O). The association with sites in wild-type bacteria in SPI-2 media compared to Δ*metJ* bacteria was still present with the more stringent DEG cutoff (see Fig. S2L) but was no longer present when analysis was restricted to upstream bases (see Fig. S2O). Finally, we leveraged the quantitative data set to examine the relationship between DMGs and DEGs in which DMGs are defined by bases completely methylated in one condition (≥99%) and hypomethylated in the other (≤89%) (see Fig. S2P to R). This revealed no additional associations, but the association with wild-type bacteria in SPI-2 media replicated once again. We conclude that for the crucial switch between SPI-1 and SPI-2 virulence gene programs, there is no association between m^6^A DNA methylation and transcriptional regulation but that specific mutants may show an association.

**TABLE 1:** Correlation of differential methylation and differential expression in wild-type *S.* Typhimurium grown in SPI-2 inducing media compared to Δ*metJ* bacteria

Genomic location	% methylated (SPI-2)		Δ% methylation^*a*^	STM14 ID	Common name	Relative location	Motif^*b*^	Log_2_ FC^*c*^
	WT	Δ*metJ*						
NC_016856.1__1217	99	88	11	STM14_0002	*thrA*	Genic	GATCA*G	–1.35
NC_016856.1__277289	99	88	11	STM14_0276	*ldcC*	Genic	GA*TC	0.89
NC_016856.1__288405	98	87	11	STM14_0289	*metN*	Genic	GA*TC	4.27
NC_016856.1__572813	93	74	19	STM14_0601	*sfbB*	Genic	CAGA*G	3.25
NC_016856.1__573231	100	90	10	STM14_0601	*sfbB*	Genic	GA*TC	3.25
NC_016856.1__573265	100	81	19	STM14_0601	*sfbB*	Genic	GA*TC	3.25
NC_016856.1__573302	100	88	12	STM14_0601	*sfbB*	Genic	GA*TC	3.25
NC_016856.1__573803	100	90	10	STM14_0602	NA^*d*^	Genic	GA*TC	3.62
NC_016856.1__861113	100	87	13	STM14_0920	*bioB*	Genic	GA*TC	–1.22
NC_016856.1__861413	100	88	12	STM14_0920	*bioB*	Genic	GA*TC	–1.22
NC_016856.1__1048140	98	88	10	STM14_1130	*ompF*	Genic	GA*TC	1.32
NC_016856.1__1423354	100	89	11	STM14_1619	*thrS*	Genic	GA*TC	–0.59
NC_016856.1__1733156	76	40	36	STM14_1975	NA	Upstream	Other	1.46
NC_016856.1__1829331	99	86	13	STM14_2086	NA	Genic	GA*TC	3.63
NC_016856.1__1831286	100	71	29	STM14_2087	*trpD*	Genic	GA*TC	3.71
NC_016856.1__1834122	100	84	16	STM14_2089	*trpB*	Genic	CAGA*G	2.13
NC_016856.1__2340851	98	81	17	STM14_2702	NA	Genic	GA*TC	2.79
NC_016856.1__2746393	100	88	12	STM14_3133	NA	Genic	GATCA*G	1.80
NC_016856.1__3270917	100	88	12	STM14_3733	*metK*	Genic	GA*TC	3.21
NC_016856.1__3272719	100	88	12	STM14_3735	NA	Genic	GA*TC	–0.82
NC_016856.1__3722412	100	90	10	STM14_4260	*asd*	Genic	CAGA*G	2.03
NC_016856.1__4334354	100	87	13	STM14_4936	*katG*	Genic	GA*TC	5.10
NC_016856.1__4417534	100	90	10	STM14_5030	*aceA*	Genic	GA*TC	2.42
NC_016856.1__4417670	100	78	22	STM14_5030	*aceA*	Genic	GA*TC	2.42
NC_016856.1__4423175	100	89	11	STM14_5035	*metH*	Genic	GA*TC	3.09
NC_016856.1__4424372	100	89	11	STM14_5035	*metH*	Genic	GA*TC	3.09

a That is, WT – Δ*metJ*.

b *, The *metJ* gene was excluded from these analyses due to artificial excision from the genome.

c That is, Δ*metJ*_SPI-2/WT_SPI-2. FC, fold change.

d NA, not applicable.

### YhdJ plays little role in *Salmonella* physiology under standard conditions important for virulence.

While our data do not support a broad, global correlation between differential methylation and differential expression under most of our tested conditions, particularly comparing wild-type bacteria grown in LB and SPI-2-inducing conditions, this does not rule out that there are discrete examples where methylation and gene expression are causally linked in our data sets. We hypothesized that such instances could be identified by combining our data on methylation and transcriptional patterns under biologically relevant conditions with data from methylase knockout mutants to reduce our search space to putative sites of regulation. We tested this hypothesis with the YhdJ (the most dynamic methylase in our data set) and Dam (the most well-studied DNA methylase in Salmonella) mutants.

**FIG 6:**
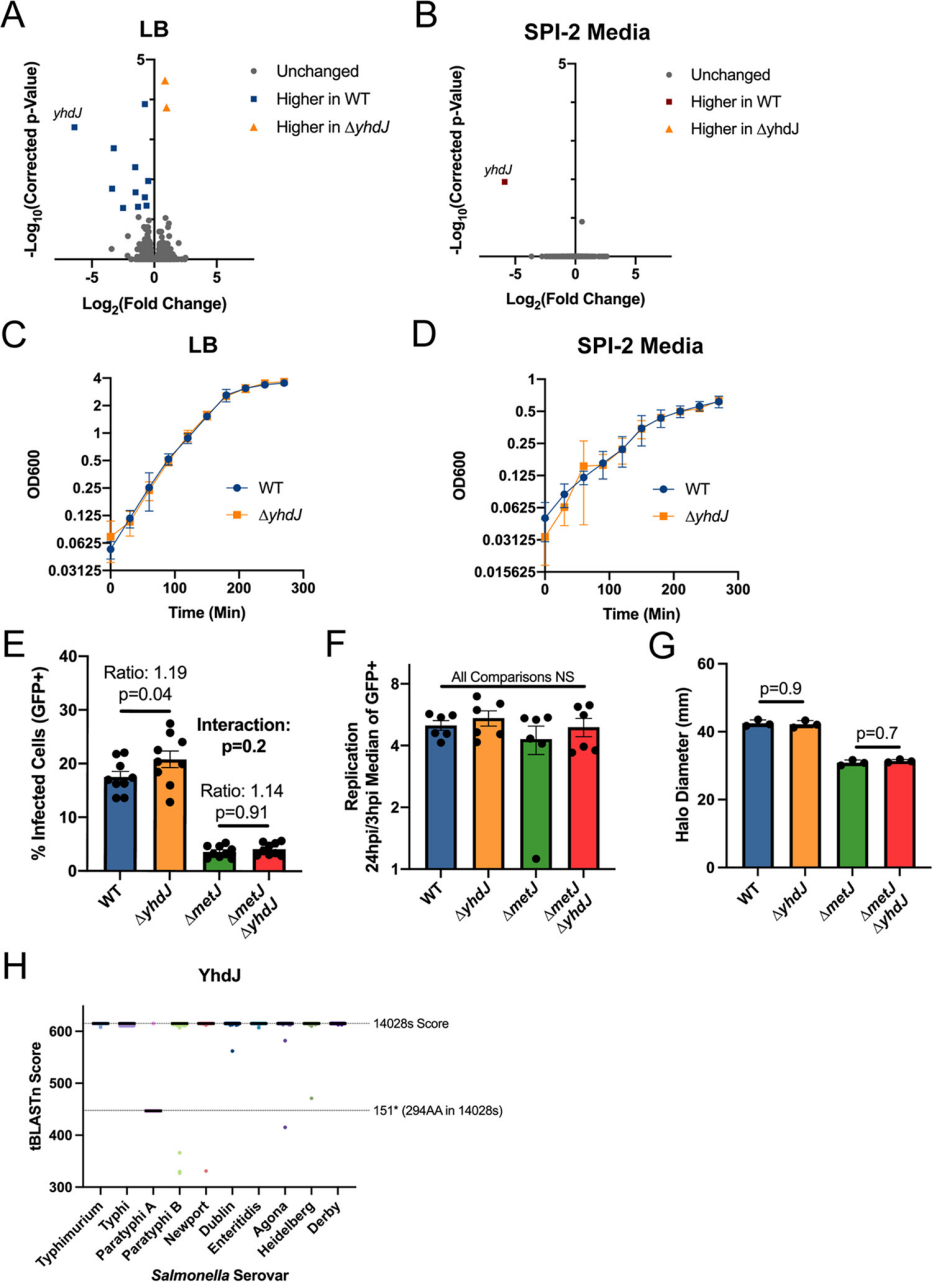
YhdJ has limited impacts on *S.* Typhimurium biology under standard laboratory conditions. (A and B). YhdJ has limited impacts on the *S.* Typhimurium transcriptome in LB (A) and SPI-2-inducing media (B). Corrected *P* values generated by calculating the FDR. (C and D) YhdJ is not required for *S.* Typhimurium growth in LB (C) or SPI-2-inducing media (D). Data from three independent experiments with time points taken every 30 min. Error bars represent the standard errors of the mean. (E and F) YhdJ is not required for *S.* Typhimurium uptake (E) or replication (F) in THP-1 monocytes. Cells were infected for 60 min with *S.* Typhimurium harboring an inducible-GFP plasmid before treatment with gentamicin. GFP was induced for 75 min before analysis by flow cytometry. The percent GFP^+^ and the median of the GFP^+^ cells were measured 3 and 24 h postinfection. Panel E shows the amount of invasion that occurred by reporting the percentage of infected cells at 3 h postinfection, and panel F shows the replication that occurred after infection by dividing the median of the GFP^+^ cells at 24 h postinfection by the median of the GFP^+^ cells at 3 h postinfection. Data are from two to three independent experiments; each dot represents an independent replicate, bars mark the mean, and error bars are the standard errors of the mean. For Panel E, data were normalized to the grand mean before plotting or performing statistics, and for panel F statistics were performed on the log transformed values. *P* values were generated by two-way analysis of variance (ANOVA) with Sidak’s multiple-comparison test. (G) YhdJ does not impact *S.* Typhimurium motility. Motility on soft agar was measured 6 h after inoculating the agar and after migration at 37°C. The data are from three independent experiments; each dot is the average of four to five technical replicates, bars mark the mean, and error bars mark the standard error of the mean. Data were normalized to the grand mean prior to plotting or performing statistics. *P* values were generated by one-way ANOVA with Sidak’s multiple-comparison test. (H) YhdJ is conserved across several Salmonella serovars. Salmonella genomes (1,000 Typhimurium, 1,000 Typhi, 1,000 Paratyphi A, 1,000 Paratyphi B, 999 Newport, 1,000 Dublin, 1,000 Enteritidis, 1,000 Agona, 1,000 Heidelberg, and 79 Derby genomes) were obtained from EnteroBase (94, 95). Genomes were combined into a single FASTA file per serovar and blasted against the *S.* Typhimurium strain 14028s YhdJ protein sequence using BLAST+ (96). The BLAST score from the top “*n*” hits were plotted, where “*n*” is the number of genomes analyzed for that serovar. A black bar represents the median. Dotted lines represent the BLAST score obtained when blasting the 14028s genome, and the score obtained from the 151* truncation prevalent in *S.* Paratyphi A serovars.

RNA-seq on wild-type and Δ*yhdJ* bacteria grown in LB or SPI-2-inducing conditions (GEO GSE185073; see Data Set S5) revealed that knocking out *yhdJ* had almost no impact on the transcriptome. Apart from *yhdJ* itself, only 12 genes were differentially expressed in LB (Fig. 6A and Table 2) despite the loss of methylation at all 513 ATGCA*T sites (see Fig. 3D), and no genes were differentially expressed under SPI-2-inducing conditions (Fig. 6B). Curiously, GO-analysis (79, 80) demonstrated the differentially expressed genes are enriched for *de novo* UMP biosynthetic processes (FDR = 6.3 × 10^−4^) and *de novo* pyrimidine nucleobase biosynthetic processes (FDR = 8.31 × 10^−4^). However, examining these genes further revealed that only two differentially expressed genes contained or were near an ATGCAT sequence (*dppA* and *pyrB*), and only *dppA* was detected to house a methylated ATGCA*T motif in our replication methylation data set (Table 2), making the mechanism of this differential expression unclear. In agreement with these findings, *yhdJ* deletion had little impact on cellular or virulence phenotypes. The Δ*yhdJ* mutation had no effect on growth in LB or SPI-2-inducing media (Fig. 6C and D), a subtle increase on the amount of observed THP-1 cell infection (Fig. 6E), no effect on replication in THP-1 cells (Fig. 6F), no effect on motility (Fig. 6G), and almost no effect on fitness in intraperitoneal (i.p.) or enteric fever models of mouse infection—though a small increase in the number of Δ*yhdJ* CFU recovered from the spleen relative to the wild type following oral gavage was observed (Table 3). Across all phenotypes, no genetic interaction between *yhdJ* and *metJ* was detected. Thus, YhdJ has very little impact on transcription or virulence associated phenotypes, and if anything, modestly impairs Salmonella virulence.

**TABLE 2:** Δ*yhdJ* differential gene expression in LB medium

Gene ID	Gene name	Log_2_ FC^*a*^ (Δ*yhdJ*/WT)	Corrected *P* value	ATGCAT motif? (relative location)	Methylated?
STM14_4375	*dppA*	–0.75	2.95E–08	Yes (Genic)	Yes
STM14_4084	*yhdJ*	–6.39	2.25E–07	No	No
STM14_5353	*pyrB*	–3.25	1.12E–06	Yes (Genic)	No
STM14_4024	*codB*	–1.52	4.51E–06	No	No
STM14_0699	*cstA*	–0.48	1.25E–05	No	No
STM14_5352	*pyrI*	–3.38	2.32E–05	No	No
STM14_0819	*NA*	0.85	3.34E–05	No	No
STM14_4495	*pyrE*	–1.49	3.84E–05	No	No
STM14_5141	*acs*	–0.74	5.74E–05	No	No
STM14_1558	*yeaG*	–0.62	0.0001	No	No
STM14_3061	*uraA*	–1.3	0.0001	No	No
STM14_0078	*carB*	–2.49	0.0001	No	No
STM14_1885	*hdeB*	0.99	0.0002	No	No

a FC, fold change.

**TABLE 3:** YhdJ does not enhance *S.* Typhimurium fitness in C57BL/6J mice

Parameter^*a*^	Δ*metJ*/WT	Δ*yhdJ*/WT	Δ*metJ *Δ*yhdJ*/WT	Δ*metJ *Δ*yhdJ*/Δ*metJ*
i.p. infection (CFU from spleen)				
No. of mice	6	8	7	7
Median (95% CI)	0.23 (0.07–0.47)	1.31 (0.60–1.84)	0.26 (0.13–0.78)	1.09 (0.000065–2.19)
*P*	0.002	0.3	0.002	0.2
	Δ*yhdJ*/WT (spleen)	Δ*yhdJ*/WT (ileum)		
Oral infection (CFU from spleen and ileum)				
No. of mice	22	21		
Median (95% CI)	1.65 (0.72–2.22)	1.154 (0.74–1.55)		
*P*	0.02	0.4		

*^a^*All mice were age and sex matched, both sexes are represented in all experiments, and all data are from at least two experiments. The median competitive index (“median”) was calculated by dividing the number colonies obtained of each genotype at 4 days postinfection. Median > 1 = numerator strain outcompeted denominator. CI, confidence interval. *P* values were calculated using a one-sample *t* test on log-transformed data.

10.1128/mbio.03464-21.10 DATA SET S5 Δ*yhdJ* RNA-seq. Download Data Set S5, XLSX file, 2.7 MB. Copyright © 2022 Bourgeois et al. 2022 Bourgeois et al. https://creativecommons.org/licenses/by/4.0/ This content is distributed under the terms of the Creative Commons Attribution 4.0 International license.

These findings suggest that YhdJ is almost entirely dispensable under the conditions tested here, despite methylating over 500 sites in the genome and methylation of its ATGCA*T motif being the most dynamic under the conditions tested. We questioned whether YhdJ may play roles outside transcription, and whether its dynamic nature is incidental. Supporting this hypothesis, an evolutionary analysis of over 9,000 strains from 10 serovars revealed that the methylase is highly conserved, with the exception of *S.* Paratyphi A, where most strains harbor a 151* truncation (Fig. 6H). This conservation paired with our RNA-seq data suggest that YhdJ methylation is likely important but is unlikely to play a large role in gene regulation.

### Integration of previous Δ*dam* literature with methylomics data reveals *stdA* hypomethylation correlates with expression in LB.

**FIG 7:**
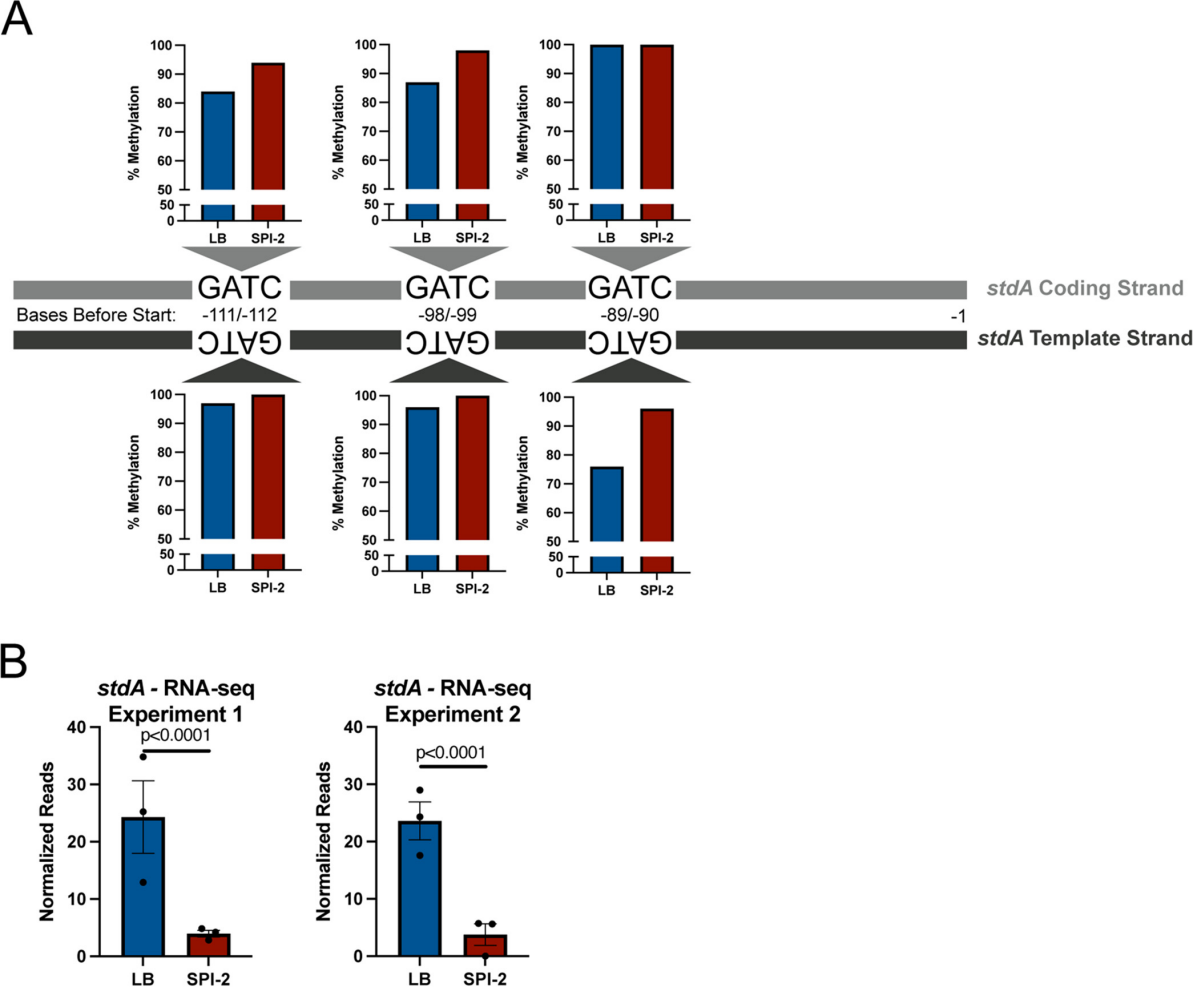
The *stdA* promoter has differential methylation after growth in LB or SPI-2-inducing media. (A) Schematic of the region upstream of *stdA*. The percent methylation values for each GATC site on both strands are graphed based on data from wild-type bacteria in methylation experiment 1. (B) *stdA* is differentially expressed in wild-type bacteria grown in LB and SPI-2-inducing media. RNA-seq experiment 1 values are from the RNA-seq experiment, including Δ*metJ* bacteria, and are listed in Data Set S4. RNA-seq experiment 2 values are from the experiment, including Δ*yhdJ* bacteria, and are listed in Data Set S5.

After demonstrating that a Δ*yhdJ* mutant could not be leveraged to identify instances where gene expression and methylation are linked, we turned to the Δ*dam* literature. Previous work on Salmonella transcriptomics revealed 17 virulence genes (making up 11 operons) that are differentially expressed between wild-type and Δ*dam S.* Typhimurium grown in LB to mid-exponential phase (31). We hypothesized that these sites may show differential methylation and expression between our LB and SPI-2 media, since Salmonella deploy radically different virulence programs across these conditions and many of these 17 virulence genes are known to be expressed specifically under only one of these two conditions (14 of 17 were DEGs comparing these two conditions in our data, and the remaining three (STM14_3654, *stdB*, and *stdC*) lacked high enough expression to analyze). In order to test this hypothesis, we searched for GATC motifs upstream (within 500 bp) of the DEGs. Interestingly, 10/11 genes or operons we examined contain GATC sites within 500 bp upstream of these genes (Table 4). However, only one gene (*stdA*) showed evidence of differential methylation under physiological conditions. Interestingly, *stdA* is the only one of these 11 genes/operons where methylation of its promoter has been extensively studied (58, 81). The three GATC sites had reduced methylation following growth in LB; however, each was only hypomethylated on a single strand (Fig. 7A). Interestingly, this hypomethylation agrees with a previous report that Dam and transcription factors compete for binding to the *stdA* promoter (58), since we also observed significantly higher expression of *stdA* in LB than in SPI-2 media (Fig. 7B). Whether this represents a mechanism by which *stdA* is turned off in SPI-2 media or is a correlated consequence of increased *stdA* transcription in LB (or vice versa) is unclear, but this finding demonstrates that the phenomenon that García-Pastor et al. describe in Δ*dam* mutants occurs naturally under biologically relevant conditions. For the other 10 virulence genes/operons that are reported to undergo differential gene expression in the *dam* mutant, we found no evidence that differential methylation plays any role during their induction during SPI-1 or SPI-2-inducing conditions.

**TABLE 4:** Percent methylation of GATC sites in experiment 1 within 500 bp upstream of Δ*dam* differentially expressed virulence genes^*a*^

Gene/operon (unlisted genes)	Base ID	% methylated in:			
		WT LB	WT SPI-2	Δ*dam* LB	Δ*dam* SPI-2
*fliC*	2060552	100	100	0	0
	2060553	100	88	0	0
	2060668	100	100	0	0
	2060669	100	100	0	0
	2060836	100	100	0	0
	2060837	100	100	0	0
*fliD* (*fliS-fliT* also in operon)	2060552	100	100	0	0
	2060553	100	88	0	0
	2060668	100	100	0	0
	2060669	100	100	0	0
*lppB*	1469508	100	100	0	0
	1469509	93	100	0	0
	1469697	100	100	0	0
	1469698	87	80	0	0
*prgH-prgI-prgJ* (*prgK* also in operon)	None	None	None	None	None
*sicA-sipB-sipC*	3051884	92	86	0	0
	3051885	98	99	0	0
	3051936	100	100	0	0
	3051937	100	100	0	0
	3052008	100	98	0	0
	3052009	99	100	0	0
	3052050	100	100	0	0
	3052051	100	100	0	0
*sipD* (*sipA-iacP* also in operon)	3048346	100	100	0	0
	3048347	99	99	0	0
*pipC* (*sopB* also in operon)	1138389	99	90	0	0
	1138390	96	97	0	0
	1138429	99	99	0	0
	1138430	100	100	0	0
	1138441	99	100	0	0
	1138442	100	100	0	0
	1138525	96	100	0	0
	1138526	98	100	0	0
*cheR* (*STM14_2332-STM14_2331-cheZ* also in operon)	2026159	90	99	0	0
	2026160	96	100	0	0
	2026385	100	100	0	0
	2026386	100	100	0	0
	2026415	100	100	0	0
	2026416	92	100	0	0
*stdA*	3211770	76	96	0	0
	3211771	100	100	0	0
	3211779	96	100	0	0
	3211780	87	98	0	0
	3211792	97	100	0	0
	3211793	84	94	0	0
*stdB-stdC* (*STM14_3655* also in operon)	3210924	100	100	0	0
	3210925	100	100	0	0
	3211283	99	99	0	0
	3211284	89	95	0	0
*STM14_3654-STM14_3653*	3206809	100	94	0	0
	3206810	100	100	0	0

a Based on reference 31.

We also attempted to integrate our data with a recent study that examined genetic heterogeneity in *S.* Typhimurium (82). We examined the 16 hypomethylated sites they identified to determine whether our data set could find the same signatures of hypomethylation. Of the seven sites we were able to find in our data set, four showed signs of hypomethylation (see Table S3). Of these four, two sites upstream of *carA* and *dgoR* showed differential methylation across our conditions; however, no consistent trends were observed with differential gene expression across our two RNA-seq data sets (see Fig. S1G to J). Thus, while we were able to observe an association of gene expression and DNA methylation for one canonical example (*stdA*) during growth in LB versus SPI-2-inducing conditions, we did not find this to be a generalizable phenomenon for other genes reported to be differentially regulated in the *dam* mutant or hypomethylated in *S.* Typhimurium.

10.1128/mbio.03464-21.5 TABLE S3 Percent methylation compared to previous hypomethylation studies. Download Table S3, DOCX file, 0.02 MB. Copyright © 2022 Bourgeois et al. 2022 Bourgeois et al. https://creativecommons.org/licenses/by/4.0/ This content is distributed under the terms of the Creative Commons Attribution 4.0 International license.

### Despite limited changes to the GA*TC methylome, *metJ* and *dam* interact to influence *S.* Typhimurium invasion and motility.

**FIG 8:**
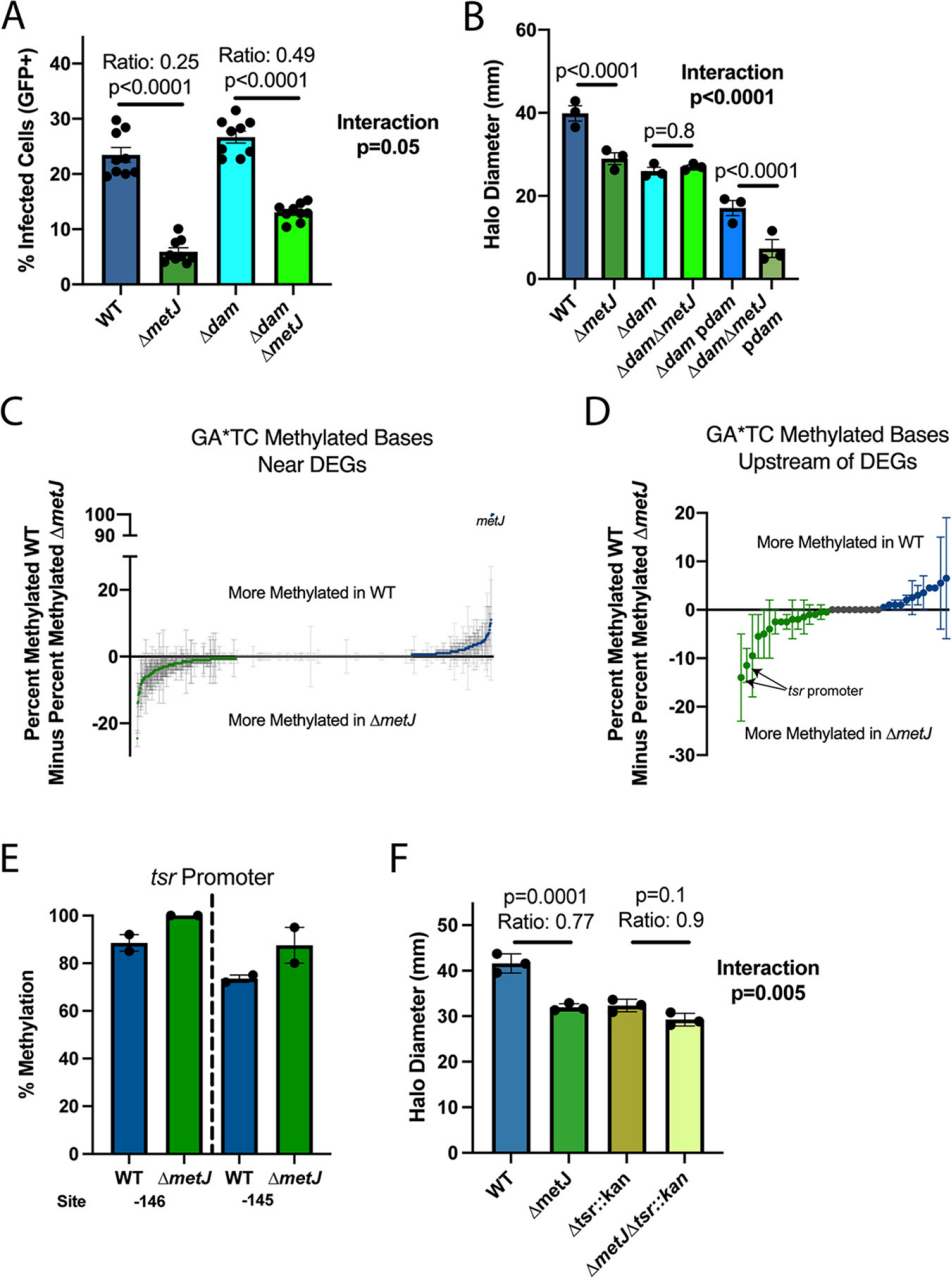
*dam* is epistatic to Δ*metJ* despite limited changes to the Δ*metJ* GA*TC methylome. (A) The impacts of Δ*metJ* on invasion partially depend on *dam*. THP-1 monocytes were infected for 60 min with *S.* Typhimurium harboring an inducible-GFP plasmid before treatment with gentamicin. GFP was induced for 75 min before analysis by flow cytometry. The percent GFP^+^ was measured at 3 h postinfection. The data are from three experiments; each dot represents an independent replicate, the bars mark the mean, and the error bars are the standard errors of the mean. (B) The impact of Δ*metJ* on motility depends entirely on *dam*. Motility on soft agar was measured 6 h after inoculating the agar and after migration at 37°C. The data are from three independent experiments; each dot is the mean of four to five technical replicates, bars mark the mean, and error bars mark the standard errors of the mean. (C and D) Quantitative analysis reveals subtle changes to the GA*TC methylome in Δ*metJ* bacteria. Each dot represents the difference average percent methylation of GA*TC bases in which the closest gene in differentially expressed (C) or GA*TC bases specifically upstream of differentially expressed genes (D), between WT and Δ*metJ* bacteria grown in LB. The data are in duplicate from the methylation experiment 1 and the replication methylation experiment, with error bars showing the error of the mean. Data from panel D are expanded in Table 4. For panels C and D, any base with greater than or less than 0 differential methylation is colored green (more methylated in Δ*metJ* bacteria) or blue (more methylated in wild-type bacteria). (E) The *tsr* promoter is modestly hypermethylated in Δ*metJ.* Percent methylation is plotted for the −146 and −145 GATC motifs from the methylation experiment 1 and the replication methylation experiment, with error bars showing the errors of the mean. Site numbering is relative to the start codon. (F) The impacts of the Δ*metJ* mutation on motility are partially *tsr* dependent. The data are from three independent experiments; each dot is the mean of four to five technical replicates, bars mark the mean, and error bars mark the standard errors of the mean. For panels A, B, and F, data were normalized to the grand mean prior to plotting or performing statistics and *P* values were generated by two-way ANOVA with Sidak’s multiple-comparison test.

Having successfully used Δ*dam* transcriptomics to identify one biologically meaningful cooccurrence of differential expression and methylation, we next attempted to leverage Δ*dam* mutants to test our hypothesis that aberrant methylation in Δ*metJ* bacteria contribute to the impact of the Δ*metJ* mutation on invasion and motility (73). Importantly, we observed a small growth defect in Δ*dam* and Δ*dam *Δ*metJ* bacteria, and so all bacteria used for these experiments were grown an extra 30 min prior to infection to standardize the growth phase used. Knocking out *dam* only modestly reduced the impact of Δ*metJ* on invasion and is therefore unlikely to be the primary mechanism by which *metJ* deletion impacts invasion (Fig. 8A). In contrast, impairment in motility caused by *metJ* deletion was completely abrogated in Δ*dam* genetic background, suggesting that *dam* and *metJ* impact motility through the same pathway (Fig. 8B). Importantly, comparing isogenic strains (wild-type versus Δ*metJ*; Δ*dam* versus Δ*dam ΔmetJ; Δdam* p*dam* versus *Δdam ΔmetJ* p*dam*) revealed that complementation of *dam* on a low-copy-number plasmid (pWSK129) restored differences between Δ*dam* and Δ*dam *Δ*metJ* bacteria, though we observed that *dam* complementation itself reduces motility further. This is likely due to modest *dam* overexpression, which has previously been reported to be a more potent inhibitor of *S.* Typhimurium 14028s motility than *dam* deletion (34).

### The motility defect of *ΔmetJ S.* Typhimurium partially depends on *tsr*.

We hypothesized that the genetic interaction between *dam* and *metJ* could signify that differential GA*TC methylation in the Δ*metJ* mutant suppresses bacterial motility. In striking contrast to this hypothesis, our combined binary data set revealed no genes that were both differentially GA*TC methylated and expressed (except for the deleted *metJ* itself) (Table 5). We next turned to our percent methylation data to examine whether a shift in methylation could explain differences in flagellar gene regulation between the two bacteria. Comparing percent methylation in both methylation data sets at all GA*TC methylated sites in which the nearest gene is differentially expressed identified 17 sites that had a ≥10% average difference in methylation between wild-type and Δ*metJ* bacteria (Fig. 8C). Because cooccurrence of differential methylation and differential expression is expected to occur frequently by chance, we sought to limit our analyses to bases most likely to impact gene expression. To do this, we restricted our search to GA*TC sites that are upstream of differentially expressed genes and found two sites of interest. Specifically, these sites are both strands of a single GATC motif upstream of the chemotaxis gene *tsr* that shows elevated methylation in Δ*metJ* bacteria (Fig. 8D and E and Table 6).

**TABLE 5:** Unique GA*TC sites between wild-type and Δ*metJ S.* Typhimurium (combined data set)

Genomic location^*a*^	Base	Genic location	Gene	Product	Differentially expressed?
Unique WT LB GA*TC sites					
None	None	None	None	None	NA^*b*^
					
Unique Δ*metJ* LB GA*TC sites					
NC_016856.1	1177765	Upstream	STM14_1293	*N*-Acetylneuraminate Epimerase	No
NC_016856.1	4289018	Downstream	STM14_4888	CDP-diacylglycerol Pyrophosphatase	No

a *, The *metJ* gene is excluded from these analyses due to artificial excision from the genome.

b NA, not applicable.

**TABLE 6:** Percent methylation differences for GA*TC motifs upstream of Δ*metJ* differentially expressed genes following growth in LB medium^*a*^

Base	Closest gene (STM14 no.)	Closest gene (common name)	% methylation (WT – *ΔmetJ*)			Gene expression	
			Methylation expt 1	Replication expt	Avg	Log_2_ FC (Δ*metJ/*WT)	Corrected *P*
4802770	STM14_5446	*tsr*	−23	−5	−14	−1.28	0.006
4802769	STM14_5446	*tsr*	−15	−8	−11.5	−1.28	0.006
2033749	STM14_2341	*flhD*	−1	−18	−9.5	−0.53	0.05
3243659	STM14_3699	*serA*	−10	−1	−5.5	1.48	5.09E–13
4415526	STM14_5029	*aceB*	−10	0	−5	1.88	6.67E–06
3335887	STM14_3821		2	−10	−4	–1.21	0.04
3400695	STM14_3893		0	−5	−2.5	−1.32	0.007
3400696	STM14_3893		−2	−3	−2.5	−1.32	0.007
4415527	STM14_5029	*aceB*	−1	−4	−2.5	1.88	6.67E–06
2060553	STM14_2378		2	−6	−2	−1.52	0.0005
3335886	STM14_3821		−4	0	−2	−1.21	0.04
571705	STM14_0600		2	−5	−1.5	4.1	1.63E–39
4415556	STM14_5029	*aceB*	−3	1	−1	1.88	6.67E–06
4802790	STM14_5446	*tsr*	−1	−1	−1	−1.28	0.006
3758504	STM14_4305		−2	1	−0.5	−1.41	6.13E–06
3846384	STM14_4392		−1	0	−0.5	3.05	3.03E–07
571704	STM14_0600		0	0	0	4.1	1.63E–39
2060669	STM14_2378		0	0	0	−1.52	0.0005
2746734	STM14_3135	*hmpA*	0	0	0	1.79	7.28E–05
2746735	STM14_3135	*hmpA*	0	0	0	1.79	7.27E–05
3243658	STM14_3699	*serA*	0	0	0	1.48	5.09E–13
3402626	STM14_3894		0	0	0	−1.23	7.87E–08
4185969	STM14_4772		0	0	0	3.17	4.96E–10
4185987	STM14_4772		0	0	0	3.17	4.96E–10
4185988	STM14_4772		0	0	0	3.17	4.96E–10
4415557	STM14_5029	*aceB*	1	0	0.5	1.88	6.67E–06
1049501	STM14_1130	*ompF*	1	1	1	1.08	4.46E–07
2060668	STM14_2378		2	0	1	–1.52	0.0005
4185970	STM14_4772		2	0	1	3.17	4.96E–10
3758505	STM14_4305		1	3	2	−1.41	6.13E–06
3846383	STM14_4392		−1	6	2.5	3.05	3.04E–07
2060552	STM14_2378		5	1	3	–1.52	0.0005
4802791	STM14_5446	*tsr*	7	0	3.5	–1.28	0.006
1049502	STM14_1130	*ompF*	5	4	4.5	1.08	4.46E–07
3402625	STM14_3894		4	5	4.5	–1.23	7.86E–08
2072703	STM14_2394	*fliJ*	15	−4	5.5	−1.55	0.006
2072704	STM14_2394	*fliJ*	19	−6	6.5	−1.55	0.006

a *, The *metJ* gene was excluded from these analyses due to artificial excision from the genome.

This hypermethylation led us to hypothesize that increased methylation upstream of *tsr* in Δ*metJ* bacteria could decrease *tsr* expression and thereby reduce motility. In line with this hypothesis, replacing *tsr* with a kanamycin resistance cassette partially ablated the ability for the Δ*metJ* mutation to cause a motility defect (Fig. 8F, interaction term *P* = 0.005). Curiously, a search for the methylation-sensitive transcription factor CRP (41) binding motif (AAATGTGATCTAGATCACATTT) in the *tsr* promoter with the MEME FIMO Tool (83) demonstrated that the hypermethylated residue lies within a putative CRP binding site. Together, these data tentatively support a model in which hypermethylation upstream of *tsr* in Δ*metJ* bacteria may contribute to the motility defect. However, additional studies are necessary to confirm a causal relationship.

### Increased methylation in the *flhDC* promoter does not contribute to the Δ*metJ* motility defect.

Given that unlike Δ*dam* (Fig. 8B**),** Δ*tsr* does not account for the entire impact of *metJ* deletion on motility (Fig. 8F, Δ*tsr*::*kanΔ metJ/*Δ*tsr*::*kan* ratio* *=* *0.9), we hypothesized that there may be additional differences in GA*TC methylation between wild-type and Δ*metJ* bacteria that impact motility. Further examination of our quantitative methylation data set (Table 6) revealed one additional plausible hypothesis: a site in the *flhDC* promoter (−278) that barely missed our 10% threshold (9.5% more methylated in Δ*metJ* bacteria). We decided to test this site as well, since FlhDC make up the master flagellar regulator, and thus modest methylated-mediated regulation of the operon could explain our findings. To test whether differential methylation of the *flhDC* promoter could explain the motility defect in Δ*metJ* bacteria, we performed site directed mutagenesis on the *S.* Typhimurium chromosome to mutate the base from GATC to GTTC. However, this mutation had no effect on motility in wild-type or Δ*metJ* bacteria (see Fig. S1K), disproving the hypothesis that this site could contribute to the Δ*dam* epistatic effect. Notably, this does not rule out that hypermethylation of this site could play a role in flagellar gene expression in other contexts but does demonstrate that it does not contribute to the Δ*metJ* motility defect.

## DISCUSSION

Here, we demonstrate that at the genome-wide level differential methylation and differential expression are not correlated. Under the critical conditions of SPI-1 or SPI-2 induction, we observed no association between DEGs and DMGs, whether examining binary changes in methylation or quantitative shifts in methylation of >10%. However, our results do demonstrate that genome-wide methylation studies of biologically relevant conditions can be integrated with data from methylase knockout mutants to identify methyl-bases that may be coupled with gene expression, as exemplified by the *stdA* and *tsr* examples. Integration of data from future methylomic studies with our publicly available data sets could reveal additional naturally occurring instances and potentially important cooccurrence of differential methylation and differential expression. As our work demonstrates, such instances will likely be challenging to identify, since they do not occur more often than expected by chance and therefore do not appear to be a general mechanism of gene regulation in Salmonella. In addition, we hope that this work encourages the generation of additional methylomic data sets under diverse and biologically relevant conditions in order to enable more intraspecies comparative methylomics.

A surprising aspect of our work was that the most differentially active methylase we observed, YhdJ, appears to have almost no impact on the *S.* Typhimurium transcriptome under standard conditions. In contrast to Dam which has known impacts on DNA and bacterial replication (10–19), YhdJ also appears to be completely nonessential for *S.* Typhimurium fitness under our growth conditions and in mice. This raises questions about the broader role of DNA methylation, and in particular YhdJ methylation, in the bacterial cell. One tantalizing hypothesis is that YhdJ plays a role in phage defense, which would have been missed studying the conditions here. Alternatively, YhdJ may contribute to physical genomic structural stability under stress conditions, similar to a proposed role for Dam during antibiotic treatment (32). Although these hypotheses could explain why YhdJ does not impact gene expression, they fail to address why we observed reproducible changes in the YhdJ methylome across different conditions. As an answer to this, we speculate that these differences are due to changes in the accessibility of YhdJ to ATGCAT motifs under the different conditions, rather than intentional targeting of YhdJ to these sites. This could be due to differences in other genomic modifications that antagonize YhdJ function, altered protein-DNA interactions that mask ATGCAT sites, and/or changes to the three-dimensional (3D) conformation of the genome that prevent interactions between YhdJ and its motif.

We propose three potential explanations for the lack of a consistent correlation between global m^6^A DNA methylation and gene expression in our data. The first is that while *S.* Typhimurium can and do use m^6^A methylation as a mechanism to promote bistability or otherwise regulate transcription, they do so sparingly. This would suggest that while the canonical examples of this are elegant (12, 21, 22, 29, 30, 36, 54–58, 82), they are rare exceptions to the general rules of *S.* Typhimurium gene regulation. While we are certainly not the first to discover individual sites of differential m^6^A methylation that do not correlate with gene expression (84, 85), this is the first analysis to demonstrate how widespread the phenomenon is in *S.* Typhimurium. The second hypothesis is that three of the four conditions tested here (wild-type or Δ*metJ* bacteria grown in LB or SPI-2-inducing media) are nonrepresentative conditions, whereas our results with the wild-type versus the *ΔmetJ* mutant in SPI-2 media are more representative of methylation’s relationship with transcription. Notably, while this is possible, these conditions were specifically chosen since they (i) are relevant to the pathogenic capacity of the bacteria, (ii) are relevant to the conditions most frequently studied in laboratory settings, or (iii) disrupt metabolic pathways directly connected to methylation. Therefore, even if methylation plays larger roles in regulating gene expression under other conditions (e.g., nutrient poor conditions at ambient temperature, following phage insult, etc.), our findings would still suggest that most observed *S.* Typhimurium phenomenon are unlikely to be linked to changes in m^6^A methylation. The third possibility is that while m^6^A is the most common modification to the *S.* Typhimurium genome, other modifications (m^5^C, phosphorothioation, etc.) may have more important impacts on gene expression.

In conclusion, through this work we have increased our understanding of the *S.* Typhimurium methylome by defining it as a highly stable system that is largely decoupled from the transcriptome at the genome-wide level. We hope that this work will serve as a reference for how to perform, analyze, and follow-up on DNA methylation studies and that it will help redefine how we think about m^6^A methylation in bacteria.

## MATERIALS AND METHODS

### Bacterial cell culture.

All Salmonella strains are derived from *S.* Typhimurium NCTC 12023 (ATCC 14028s) and are included in Table S1. All plasmids are included in Table S1. Chromosomal knockouts were generated by lambda-red recombineering (86). Site-directed mutagenesis of the chromosome was performed using a modified version of lambda-red recombineering, as previously described (87). Complementation plasmids were generated by cut and paste cloning using the pWSK129 plasmid (88). For all experiments, bacteria were maintained on LB (BD, Miller formulation) agar plates, grown in LB media overnight at 37°C at 250 rpm, and subcultured the following morning prior to experiments. The SPI-2-inducing media is the low phosphate and magnesium (LPM) media from Coombes et al. (59) and contains 5 mM KCl, 7.5 mM (NH_4_)_2_SO_4_, 0.5 mM K_2_SO_4_, 38 mM glycerol (0.3% [vol/vol]), 0.1% casein hydrolysate, 8 μM MgCl_2_, 337 μM K_2_HPO_4_ (pH 5.8), 80 mM MES (pH 5.8), with the final solution pH equal to 5.8. Propagation of temperature-sensitive plasmids occurred at 30°C and were cured at 42°C. Ampicillin was added to media at 100 μg/mL, kanamycin at 50 μg/mL, and apramycin at 100 μg/mL.

10.1128/mbio.03464-21.3 TABLE S1 Bacterial strains and plasmids used in this study. Download Table S1, DOCX file, 0.02 MB. Copyright © 2022 Bourgeois et al. 2022 Bourgeois et al. https://creativecommons.org/licenses/by/4.0/ This content is distributed under the terms of the Creative Commons Attribution 4.0 International license.

### Mammalian cell culture.

THP-1 monocytes from the Duke Cell Culture Facility were cultured at 37°C in 5% CO_2_ in RPMI 1650 media (Invitrogen) supplemented with 10% heat-inactivated fetal bovine serum, 2 μM glutamine, 100 U/mL penicillin-G, and 100 mg/mL streptomycin. Cells used for Salmonella gentamicin protection assays were grown in antibiotic-free media 1 h prior to infection.

### Sample preparation for SMRT-Seq.

*S.* Typhimurium were grown overnight, washed once, and subcultured 1:33 in LB for 2 h and 45 min to induce SPI-1 expression or 1:50 in SPI-2-inducing media for 4 h in order to induce SPI-2 expression. A total of 2 × 10^9^ bacteria were pelleted, and DNA was extracted using a DNeasy blood and tissue kit (Qiagen). The optional RNase step in the protocol was performed to remove contaminating RNA according to the manufacturer’s instructions. DNA was stored at 4°C until library preparation. Multiplexed SMRTbell libraries for sequencing on a PacBio Sequel system were prepared from 1 μg of each microbial gDNA sample. Shearing of gDNA was performed using g-TUBE and centrifugation at 2,029 × *g* for 2 min to achieve a target mode size of 10 to 15 kb.

SMRTbell libraries were then prepared using the SMRTbell Express Template Prep kit 2.0. Two pools of eight indexed libraries were prepared. Each pool was then sequenced on a PacBio Sequel SMRTcell using sequencing chemistry 3.0 and 10-h movie length.

### Sample preparation for RNA-Seq.

*S.* Typhimurium were grown overnight, washed once, and subcultured 1:33 in LB for 2 h and 45 min or 1:50 in SPI-2-inducing media for 4 h. A total of 2 × 10^9^ bacteria were pelleted at 5,000 × *g* for 5 min and resuspended in RNAprotect bacterial reagent (Qiagen) in order to stabilize transcripts. After 5 min, bacteria were repelleted, and resuspended in 200 μL of TE buffer containing lysozyme (15 mg/mL) and 20 μL of proteinase K. Bacteria were vortexed every 2 min for 15 min. Then, 700 μL of β-mercaptoethanol-containing RLT buffer was added. After vortexing, 500 μL of 96% ethanol was added, and the solution was mixed and applied to a RNeasy extraction column (Qiagen). The remainder of the RNeasy protocol was followed according to the manufacturer’s instructions. After RNA isolation, 3 to 6 μg of RNA was treated with Turbo DNase (Thermo-Fisher) according to the manufacturer’s instructions, with the exception that two successive 30-min DNase treatments were performed. To remove DNase after treatment, the solution was mixed with 350 μL of β-mercaptoethanol-containing RLT buffer, and then 700 μL of 96% ethanol was added. The mixture was then added to a RNeasy MinElute column (Qiagen), and RNA was reisolated according to the manufacturer’s instructions.

RNA samples QC was performed with an Agilent fragment analyzer and a Qubit assay on the Perkin-Elmer Victor X2. An Illumina TruSeq Stranded total RNA-Seq kit combined with a Ribo-Zero rRNA removal kit (bacteria) was used to prepare total RNA-seq libraries. Total RNA was first depleted of the rRNA using biotinylated probes that selectively bind to rRNA molecules. The rRNA depleted RNA was then reverse transcribed. During the second-strand synthesis, the cDNA:RNA hybrid is converted into to double-stranded cDNA (dscDNA) and dUTP incorporated into the second cDNA strand, effectively marking the second strand. Illumina sequencing adapters were then ligated to the dscDNA fragments and amplified to produce the final RNA-seq library. The strand marked with dUTP is not amplified, allowing strand specificity sequencing. Libraries were indexed using a dual indexing approach allowing for multiple libraries to be pooled and sequenced on the same sequencing flow cell of an Illumina MiSeq sequencing platform. Before pooling and sequencing, fragment length distribution and library quality were first assessed on a fragment analyzer using a high-sensitivity DNA kit (Agilent Technologies). All libraries were then pooled in equimolar ratio and sequenced. Sequencing was done using 50-bp single-end reads. Once generated, sequence data were demultiplexed and Fastq files generated using Bcl2Fastq conversion software from Illumina.

### SMRT-seq mapping and m^6^A analysis.

m^6^A methylation calls were performed using the pbsmrtpipe base modification and motif detection pipeline (Smrtlink v7.0.1.66975) with Salmonella enterica serovar Typhimurium strain 14028s (ASM2216v1) as the reference genome. For sites at or above 50× coverage, sites with a phred-based quality score greater than 40 were marked as “1,” for strong evidence of methylation; sites with ≥50× coverage but below QV40 were marked as “0,” for unlikely to be methylated. For sites below 50× coverage, methylation status was not estimated. Assigning methylated bases to motif(s) was performed by comparing the context of the base to known or identified motifs using Microsoft Excel. Motif enrichment was calculated by dividing the frequency of the motif in a given subset (e.g., frequency of the motif in bases only methylated in bacteria grown in LB) and dividing by the frequency of the motif in condition tested (e.g., frequency of the motif among all methylated bases in bacteria grown in LB). Additional methyl-bases were detected on the pWSK29 plasmid harbored in these strains; however, we did not include these bases in our analyses since this plasmid is not involved in the natural lifestyle of *S.* Typhimurium.

### RNA-seq analysis and integration with methylomics.

RNA-seq data were processed using the TrimGalore toolkit (http://www.bioinformatics.babraham.ac.uk/projects/trim_galore) which employs Cutadapt (89) to trim low-quality bases and Illumina sequencing adapters from the 3′ end of the reads. Only reads that were 20 nucleotides or longer after trimming were kept for further analysis. Reads were mapped to the ASM2216v1 version of the Salmonella enterica strain 14028S genome and transcriptome (90) using the STAR RNA-seq alignment tool (91). Reads were kept for subsequent analysis if they mapped to a single genomic location. Gene counts were compiled using the HTSeq tool (http://www-huber.embl.de/users/anders/HTSeq/). Only genes that had at least 10 reads in any given library were used in subsequent analysis. Normalization and differential expression were carried out using the DESeq2 (92) Bioconductor (93) package with the R statistical programming environment (https://www.R-project.org/). The FDR was calculated to control for multiple hypothesis testing.

Integration of methylomics and RNA-seq analysis occurred in three steps. First, a list of genes present in both analyses was generated. Second, rates of differential expression among (i) the entire list of genes present in both analyses and (ii) differentially methylated genes were called as genes containing 1+ base that was methylated in one condition but not another. Third, expected (frequency of differential expression in the entire list of genes present in both analyses multiplied by the frequency of differential methylation multiplied by the total number of genes in the analysis) and observed differentially methylated and differentially expressed genes were compared. A Fisher exact test was used to determine whether there were statistically significant associations between differential methylation and differential expression.

### Analysis of *yhdJ* across *Salmonella* genomes.

In order to analyze conservation of *yhdJ* across the Salmonella enterica genomes, 9,078 genomes (1,000 Typhimurium, 1,000 Typhi, 1,000 Paratyphi A, 1,000 Paratyphi B, 999 Newport, 1,000 Dublin, 1,000 Enteritidis, 1,000 Agona, 1,000 Heidelberg, and 79 Derby genomes) were obtained from the EnteroBase repository (94, 95). Serovars examined here were specifically chosen to test for conservation among a diverse group of Salmonella. The specific strains were randomly selected and represented a variety of sources (human, agricultural animal, avian, reptiles, environment, etc.) within serovars, when possible. After downloading the genomes, all genomes of a given serovar were concatenated into a single FASTA file and used for analysis with the BLAST+ command line software (96). The 14028s YhdJ protein sequence was used as query for the pBLASTn program. To determine conservation, the program produced BLAST scores for “*n*” sequences, where *n* = the number of strains tested within each serovar. The BLAST scores were then plotted relative to the BLAST score obtained using the 14028s genome.

### GO-term analysis.

All GO-terms were generated using the Gene Ontology Resource (http://geneontology.org/) (79, 80). A PANTHER overrepresentation test was run using the Salmonella Typhimurium GO biological process reference, the test used the Fisher exact test, and the correction was based on a calculated false discovery rate. All calculations were run automatically though the web portal software. Any gene that was not present in the GO-term database was “unmapped” and excluded from the analysis.

### Growth curves.

*S.* Typhimurium were grown overnight in LB, subcultured 1:50 into 5 mL of either LB or SPI-2-inducing media, and grown at 37°C at 250RPM. OD600 measurements were taken every 30 min using a spectrophotometer (Pharmacia Biotech Novaspec II).

### Gentamicin protection assay.

Invasion and replication were measured as previously described (97–99). Briefly, bacteria were grown overnight, subcultured 1:33 into 1 mL of LB, and grown for 2 h and 45 min or until all strains entered late-log phase growth (OD_600_ = 1.5 to 2.0) at 37°C with 250 rpm. For any experiment using Δ*dam* bacteria, all bacteria were grown an extra 30 min (3 h and 15 min) so that the Δ*dam* and Δ*dam* Δ*metJ* mutants reached late-exponential-phase growth. A total of 100,000 THP-1 monocytes in antibiotic-free media were then infected by *S.* Typhimurium (MOI of 5). At 1 h postinfection, cells were treated with gentamicin (50 μg/mL), and IPTG (isopropyl-β-d-thiogalactopyranoside) was added 2 h postinfection to induce bacterial GFP expression. At 3 h and 15 min postinfection, cells were read by using a Guava Easycyte Plus flow cytometer (Millipore). At 22 h and 45 min postinfection, IPTG was added to remaining wells to induce GFP, and at 24 h postinfection the cells were quantified by flow cytometry. The percent host cell invasion was determined by quantifying the number of GFP^+^ cells 3 h and 15 min postinfection, and replication was assessed by determining the ratio of the median intensity of GFP^+^ cells at 24 h postinfection divided by the median of the GFP^+^ cells at 3 h and 15 min postinfection.

### Motility assays.

All strains were cultured overnight in LB, subcultured 1:33 into LB, and grown for 2 h and 45 min at 37°C with 250 rpm. A pipette tip was used to puncture and deliver 2 μL of *S.* Typhimurium into the center of a 0.3% LB agar plate. Plates were incubated at 37°C for 6 h before the halo diameter was quantified.

### Murine competitive index experiments.

Mouse studies were approved by the Duke Institutional Animal Care and Use Committee and adhere to the *Guide for the Care and Use of Laboratory Animals* of the National Institutes of Health. All experiments were performed with age- and sex-matched C57BL/6J (7- to 14-week-old) mice. Bacteria were grown overnight, subcultured 1:33, and grown for 2 h and 45 min at 37°C at 250 rpm. The bacteria were then washed and resuspended in phosphate-buffered saline. Inoculua were confirmed by plating for CFU. For oral infections, mice were fasted for 12 h before infection and given 100 μL of a 10% sodium bicarbonate solution by oral gavage 30 min before infection. Mice then received a 1:1 mixture of two *S.* Typhimurium strains containing either pWSK29 (AmpR) or pWSK129 (KanR) (88), totaling 10^8^ CFU in 100 μL, by oral gavage. For i.p. infections, mice were injected with a 1:1 mixture of two *S.* Typhimurium strains, totaling 10^3^ CFU in 100 μL, into the i.p. space. For both models, tissues were harvested 4 days postinfection, homogenized, and plated on LB agar containing either ampicillin or kanamycin. The competitive index was calculated as follows: (no. of strain A CFU in tissue/no. of strain B CFU in tissue)/(no. of strain A CFU in inoculum/no. of strain B CFU in inoculum). Statistics were calculated by log transforming this ratio from each mouse and comparing to an expected value of 0 using a one-sample *t* test.

### RT-qPCR.

RNA was harvested as described above and used to create cDNA using the iScript cDNA synthesis kit (Bio-Rad Laboratories). qPCR was performed using the iTaq Universal SYBR green Supermix (Bio-Rad Laboratories). Next, 10-μL reactions contained 5 μL of the supermix, a final 500 nM concentration of each primer, and 2 μL of cDNA. Reactions were run on a QuantStudio 3 Thermo Cycler. The cycling conditions were as follows: (i) 95°C for 30 s, (ii) 40 cycles of 95°C for 15 s and 60°C for 60 s, and then (iii) 60°C for 60 s. A melting curve was determined in order to verify single PCR products. The comparative threshold cycle (*C_T_*) method was used to quantify transcripts, with the ribosomal *rrs* gene serving as the endogenous control. The fold change represents 2^–ΔΔ^*^CT^*. Oligonucleotides are listed in Table S2.

10.1128/mbio.03464-21.4 TABLE S2 Oligonucleotides used in this study. Download Table S2, DOCX file, 0.02 MB. Copyright © 2022 Bourgeois et al. 2022 Bourgeois et al. https://creativecommons.org/licenses/by/4.0/ This content is distributed under the terms of the Creative Commons Attribution 4.0 International license.

### Western blotting.

*flhC* was tagged with the 3xFLAG tag using recombineering as previously described (100). *S.* Typhimurium bacteria were grown overnight in LB, subcultured 1:33 in LB at 37°C and 250 rpm until reaching late log phase (OD_600_ = 1.5 to 2.0), and then pelleted by centrifugation at 6,000 × *g* for 5 min. Pellets were resuspended in 2× Laemmli buffer (Bio-Rad) with 5% 2-mercaptoethanol and boiled for 10 min, and lysates were run on Mini-Protean TGX stain-free gels (Bio-Rad). After electrophoresis, the gels’ total protein dye was activated by a 5-min UV exposure. After transfer onto Immun-Blot low-fluorescence polyvinylidene difluoride membrane (Bio-Rad) using a Hoefer TE77X, blots were probed using an anti-FLAG M2 antibody (Sigma, catalog no. F3165). A florescent secondary antibody (LI-COR IRDye) was used to detect bands on a LI-COR Odyssey Classic. Band intensity was quantified using LI-COR Odyssey Imaging System Software v3.0. Total protein was detected by 30 s of UV exposure and quantified using Fiji (101). The graphed relative signal was calculated as follows: (FLAG band intensity/total protein)/(FLAG band intensity in wild-type *flhC:FLAG3×* bacteria/total protein in wild-type *flhC:FLAG3×* bacteria).

### Statistical analyses.

Statistics were performed using GraphPad Prism 9 or Microsoft Excel, except where otherwise noted. Where noted, interexperimental noise was removed from gentamicin protection assays and motility assays prior to data visualization or statistical analysis by standardizing data to the grand mean by multiplying values within an experiment by a constant (the average of all experiments divided by the averages of specific experiments). All statistical tests corresponding to reported *P* values are described in the appropriate figure legends.

### Data availability.

All sequencing data are available in the NCBI’s Gene Expression Omnibus (GEO) (102) Super Series (GSE185077). This includes both SMRT-seq experiments (GSE185578 and GSE185501), as well as both RNA-seq experiments (GSE185072 and GSE185073). All biological resources are available upon request to Dennis Ko.
