# Supplementary figures and images for: Integration of the Salmonella Typhimurium Methylome and Transcriptome Reveals That DNA Methylation and Transcriptional Regulation Are Largely Decoupled under Virulence-Related Conditions
Source: mBio. 2022 Jun 6;13(3):e03464-21. doi: 10.1128/mbio.03464-21 (PMC9239280; doi:10.1128/mbio.03464-21)

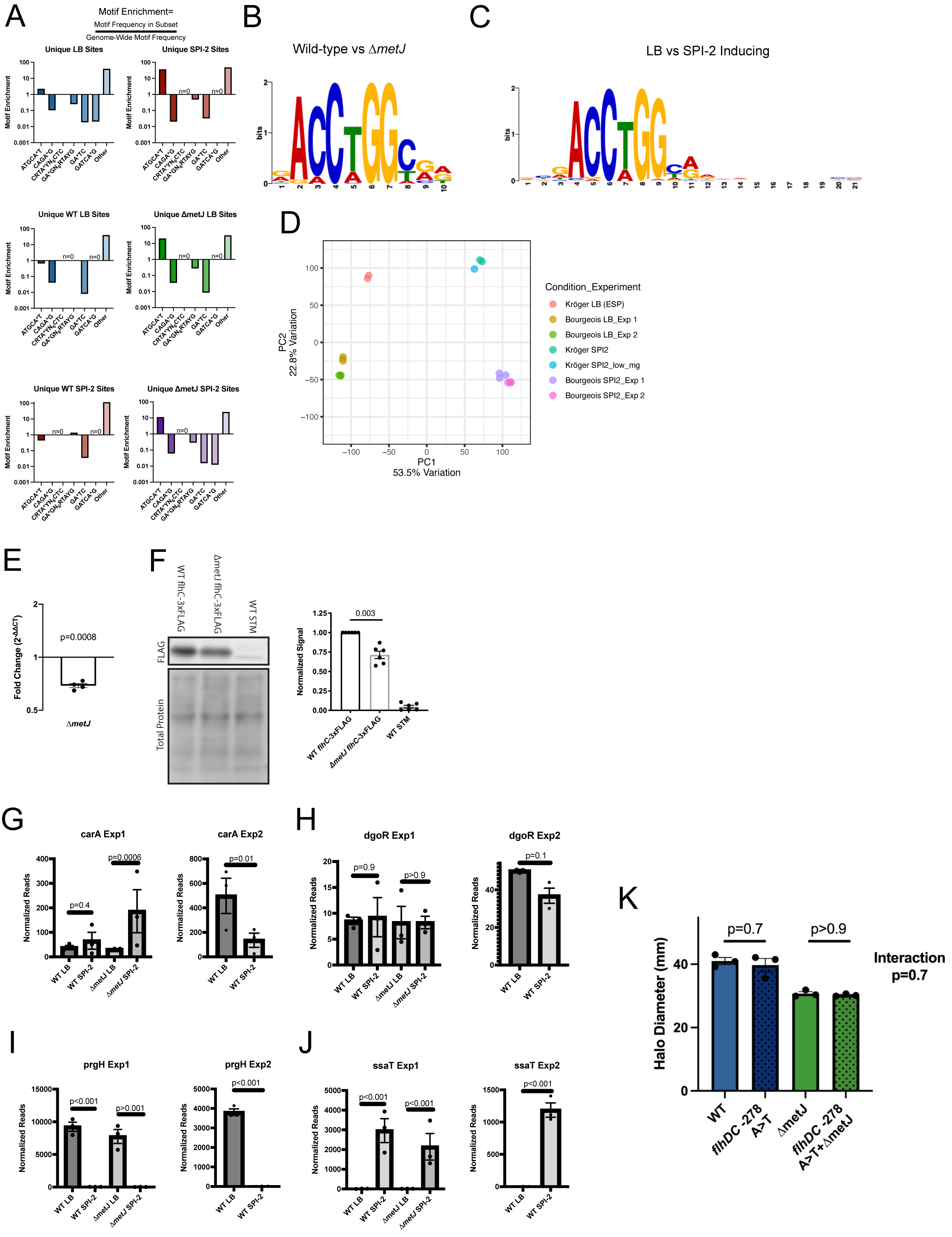

Supplement: FIG S1 [file mbio.03464-21-s0009.tif]

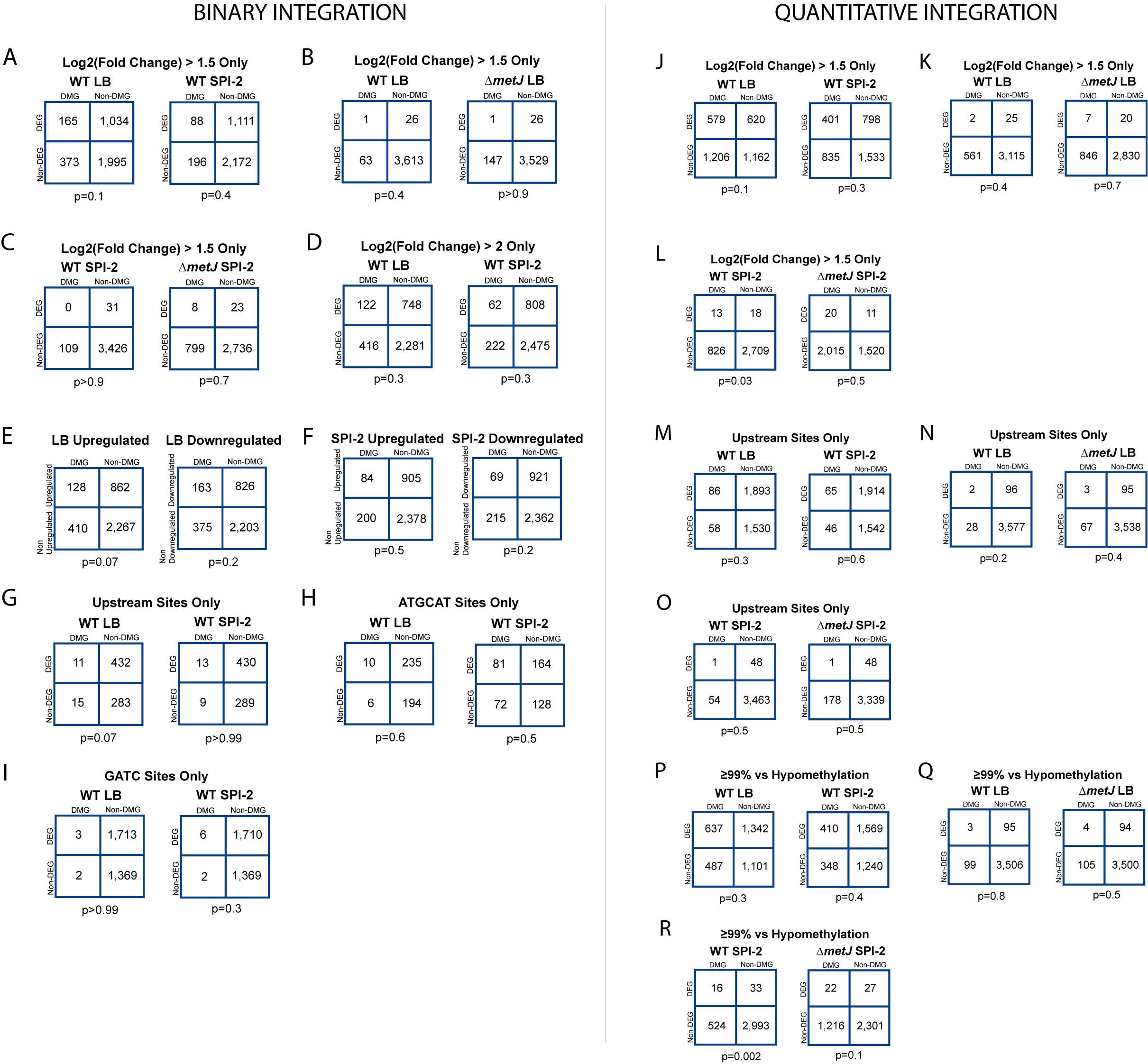

Supplement: FIG S2 [file mbio.03464-21-s0010.tif]
