# Supplementary material for: Integration of the Salmonella Typhimurium Methylome and Transcriptome Reveals That DNA Methylation and Transcriptional Regulation Are Largely Decoupled under Virulence-Related Conditions
Source: mBio. 2022 Jun 6;13(3):e03464-21. doi: 10.1128/mbio.03464-21 (PMC9239280; doi:10.1128/mbio.03464-21)
Supplement: TABLE S3 [file mbio.03464-21-s0008.docx]

| **Supplemental Table 3: Percent methylation compared to previous hypomethylation studies** | | | | | | | | |
| --- | --- | --- | --- | --- | --- | --- | --- | --- |
| **^Reproduced and Adapted from Sánchez-Romero *et al*. (1)** | | | | | | | | |
| **Gene^** | **Gene Product^** | **Number of GATCs^** | **Number of under-methylated GATCs^** | **Position(s) of GATC undermethylated A(s)^** | **Average % Methylation in wild-type bacteria grown in LB in this study (Combined Dataset)**  ***N/A = Could not find in dataset** | **Average % Methylation in ∆*metJ* bacteria grown in LB in this study (Combined Dataset)**  ***N/A = Could not find in dataset** | **% Methylation in wild-type SPI-2 media (Experiment 1)**  ***N/A = Count not find in dataset** | **% Methylation in ∆metJ SPI-2 media (Experiment 1)**  ***N/A = Count not find in dataset** |
| carA | Carbamoyl-phosphate synthase small chain | 2 | 2 | −511, −206 | **-511** = 52%  **-206** = 45.5% | **-511** = 49.5%  **-206** = 45% | **-511** = 49%  **-206** = 88% | **-511** = 69%  **-206** = 99% |
| dgoR | Galactonate operon transcriptional repressor | 1 | 1 | −147 | **-147** = 0% | **-147** =15.5% | **-147** = 63% | **-147** = 73% |
| ftnB | Ferritin-like protein | 4 | 2 | −340, −156 | **-340** = **N/A***  **-156** = 100% | **-340** = **N/A***  **-156** = 96.5% | **-340** = **N/A***  **-156** = 100% | **-340** = **N/A***  **-156** = 100% |
| gtr | O-antigen glycotransferase | 4 | 2 | −69, −56 | **N/A*** | **N/A*** | **N/A*** | **N/A*** |
| holA | DNA polymerase III, delta subunit | 5 | 1 | 4 | **+4** = 98.5% | **+4** = 97.5% | **+4** = 100% | **+4** = 100% |
| nanA | N-acetylneuraminate lyase | 8 | 1 | −58 | **-59*** = 13%  *No -58 GATC site in our mapping | **-59*** = 30.5%  *No -58 GATC site in our mapping | **-59*** = 0%  *No -58 GATC site in our mapping | **-59*** = 0%  *No -58 GATC site in our mapping |
| opvAB | O-antigen chain length regulation | 4 | 2 | −178, −105 | **N/A*** | **N/A*** | **N/A*** | **N/A*** |
| slrA | Glucitol/sorbitol-specific enzyme IIC component | 2 | 1 | −86 | **N/A*** | **N/A*** | **N/A*** | **N/A*** |
| ssaN | Type III secretion ATP synthase | 2 | 1 | −202 | **-202** = 100% | **-202** = 100% | **-202** = 100% | **-202** = 100% |
| STM1290 | N-acetylmannosamine-6-phosphate-2-epimerase | 2 | 1 | −465 | **N/A*** | **N/A*** | **N/A*** | **N/A*** |
| STM2047 | Hypothetical protein | 3 | 1 | −68 | **N/A*** | **N/A*** | **N/A*** | **N/A*** |
| STM3726 | Putative mannitol dehydrogenase | 3 | 1 | −68 | **N/A*** | **N/A*** | **N/A*** | **N/A*** |
| STM4889 | Putative Na^+^/galactosidase symporter | 4 | 1 | −171 | **N/A*** | **N/A*** | **N/A*** | **N/A*** |
| STM5047 | Putative cytoplasmic protein | 3 | 1 | −108 | **N/A*** | **N/A*** | **N/A*** | **N/A*** |
| STM5308 | Sugar transporter | 3 | 1 | −66 | **N/A*** | **N/A*** | **N/A*** | **N/A*** |
| yihU | Hypothetical oxidoreductase | 2 | 1 | −66 | **N/A*** | **N/A*** | **N/A*** | **N/A*** |

1. Sanchez-Romero, M.A., Olivenza, D.R., Gutierrez, G. and Casadesus, J. (2020) Contribution of DNA adenine methylation to gene expression heterogeneity in Salmonella enterica. *Nucleic Acids Res*, **48**, 11857-11867.
