# Supplementary material for: Integration of the Salmonella Typhimurium Methylome and Transcriptome Reveals That DNA Methylation and Transcriptional Regulation Are Largely Decoupled under Virulence-Related Conditions
Source: mBio. 2022 Jun 6;13(3):e03464-21. doi: 10.1128/mbio.03464-21 (PMC9239280; doi:10.1128/mbio.03464-21)
Supplement: TABLE S1 [file mbio.03464-21-s0006.docx]

| **Supplemental Table 1: Bacterial strains and Plasmids used in this study** | | | |
| --- | --- | --- | --- |
| **Bacterial Strains** | | | |
| Identifier | Genotype | Plasmid | Antibiotic Resistance |
| DCK543 | *S.* Typhimurium NTCC 12023 (ATCC 14028s) |  |  |
| DCK545 | ∆*metJ* |  |  |
| DCK546 | *S.* Typhimurium NTCC 12023 (ATCC 14028s) | pWSK29 | Ampicillin |
| DCK547 | ∆*metJ* | pWSK29 | Ampicillin |
| DCK571 | *S.* Typhimurium NTCC 12023 (ATCC 14028s) | p67GFP3.1 | Ampicillin |
| DCK573 | ∆*metJ* | p67GFP3.1 | Ampicillin |
| DCK574 | *S.* Typhimurium NTCC 12023 (ATCC 14028s) | pWSK129 | Kanamycin |
| DCK576 | ∆*metJ* | pWSK129 | Kanamycin |
| DCK707 | ∆*dam* |  |  |
| DCK708 | ∆*dam*∆*metJ* |  |  |
| DCK711 | ∆*dam* | p67GFP3.1 | Ampicillin |
| DCK712 | ∆*dam*∆*metJ* | p67GFP3.1 | Ampicillin |
| DCK857 | ∆*dam* | pWSK29 | Ampicillin |
| DCK858 | ∆*dam*∆*metJ* | pWSK29 | Ampicillin |
| DCK859 | ∆*dam* | pWSK129 | Kanamycin |
| DCK860 | ∆*dam*∆*metJ* | pWSK129 | Kanamycin |
| DCK874 | ∆*dam* | pWSK129::*dam* | Kanamycin |
| DCK875 | ∆*dam*∆*metJ* | pWSK129::*dam* | Kanamycin |
| DCK934 | ∆*yhdJ* |  |  |
| DCK935 | ∆*metJ*∆*yhdJ* |  |  |
| DCK936 | ∆*yhdJ* | p67GFP3.1 | Ampicillin |
| DCK937 | ∆*metJ*∆*yhdJ* | p67GFP3.1 | Ampicillin |
| DCK938 | ∆*yhdJ* | pWSK29 | Ampicillin |
| DCK1015 | ∆*metJ*∆*yhdJ* | pWSK29 | Ampicillin |
| DCK939 | ∆*metJ*∆*yhdJ* | pWSK129 | Kanamycin |
| DCK865 | *flhC::3xFLAG* |  |  |
| DCK866 | *∆metJ*; *flhC::3xFLAG* |  |  |
| DCK1152 | *flhDC* promoter -278 A>T |  |  |
| DCK1155 | ∆*metJ*; *flhDC* promoter -278 A>T |  |  |
| DCK1157 | *∆tsr::Kan^R^* |  | Kanamycin |
| DCK1158 | ∆*metJ*∆*tsr::Kan^R^* |  | Kanamycin |
| **Plasmids** | | | |
| **Identifier** | **Plasmid** | **Antibiotic Resistance** | **Source** |
| DCK482 | pWSK29 | Ampicillin | (1) |
| DCK827 | pWSK129 | Kanamycin | (1) |
| DCK22 | P67GFP3.1 | Ampicillin | (2) |
| CS946 | pKD4 | Kanamycin | (3) |
| DCK599 | pKD46 | Ampicillin | (3) |
| CS943 | pCP20 | Ampicillin | (3) |
| DCK494 | pSUB11 | Ampicillin, Kanamycin | (4) |
| DCK1094 | pREDTKI | Kanamycin | (5) |
| DCK1095 | pMDIAI | Ampicillin | (5) |
| DCK1096 | pKSI-1 | Ampicillin | (5) |
| DCK855 | pWSK129::*dam* (includes 180 bases upstream and 5 bases downstream) | Kanamycin | This work |

1. Wang, R.F. and Kushner, S.R. (1991) Construction of versatile low-copy-number vectors for cloning, sequencing and gene expression in Escherichia coli. *Gene*, **100**, 195-199.

2. Pujol, C. and Bliska, J.B. (2003) The ability to replicate in macrophages is conserved between Yersinia pestis and Yersinia pseudotuberculosis. *Infect Immun*, **71**, 5892-5899.

3. Datsenko, K.A. and Wanner, B.L. (2000) One-step inactivation of chromosomal genes in Escherichia coli K-12 using PCR products. *Proc Natl Acad Sci U S A*, **97**, 6640-6645.

4. Uzzau, S., Figueroa-Bossi, N., Rubino, S. and Bossi, L. (2001) Epitope tagging of chromosomal genes in Salmonella. *Proc Natl Acad Sci U S A*, **98**, 15264-15269.

5. Yang, J., Sun, B., Huang, H., Jiang, Y., Diao, L., Chen, B., Xu, C., Wang, X., Liu, J., Jiang, W. *et al.* (2014) High-efficiency scarless genetic modification in Escherichia coli by using lambda red recombination and I-SceI cleavage. *Appl Environ Microbiol*, **80**, 3826-3834.
