# Supplementary material for: Integration of the Salmonella Typhimurium Methylome and Transcriptome Reveals That DNA Methylation and Transcriptional Regulation Are Largely Decoupled under Virulence-Related Conditions
Source: mBio. 2022 Jun 6;13(3):e03464-21. doi: 10.1128/mbio.03464-21 (PMC9239280; doi:10.1128/mbio.03464-21)
Supplement: TABLE S2 [file mbio.03464-21-s0007.docx]

| **Supplemental Table 2: Oligonucleotides used in this study** | | | |
| --- | --- | --- | --- |
| Name | Sequence (5’ 🡪 3’) | Purpose | Source |
| *flhD* Forward | TGTTCCGCCTCGGTATCAAC | qPCR | (1) |
| *flhD* Reverse | CGCGAATCCTGAGTCAAACG | qPCR | (1) |
| *dam* KD4 Forward | CTTTCTCCACAGCCGGAGAAGGTGTAATTAGTTAGTCAGCGTGTAGGCTGGAGCTGCTTC | Kanamycin Cassette | This study |
| *dam* KD4 Reverse | GGGGCAATCAAATACTGTTTCATCCGCTTCTCCTTGAGAACATATGAATATCCTCCTTAG | Kanamycin Cassette | This study |
| *yhdJ* KD4 Forward | GGGAAGCGCC TTTTTTATACGCATCACATG GAATTTGGTCGTGTAGGCTGGAGCTGCTTC | Kanamycin Cassette | This study |
| *yhdJ* KD4 Reverse | ACATGAAATTTTGACGCTGAAAAGCGGACTTACAATGCTTCATATGAATATCCTCCTTAG | Kanamycin Cassette | This study |
| *tsr* KD4 Forward | AGGCCGAAAATTCTGTATCTGTCTAGCGGAAAGAGAAAACGTGTAGGCTGGAGCTGCTTC | Kanamycin Cassette | This study |
| *tsr* KD4 Reverse | CCTACGGTCGTCTGTAGGCCGACTGTTCACCACTACGCCCCATATGAATATCCTCCTTAG | Kanamycin Cassette | This study |
| *flhC* pSUB11 Forward | TATTCCACAACTGCTGGATGAACAGATCGAACAGGCTGTTGACTACAAAGACCATGACGG | 3xFLAG tag | This study |
| *flhC* pSUB11 Reverse | TGACTTACCGCTGCTGGAGTGTTTGTCCACACCGTTTCGGCATATGAATATCCTCCTTAG | 3x FLAG tag | This study |
| *flhD* pMDIAI Forward | GGTTATTAATTAAACAAAGTAAAAGCCATGCTGATGGGTTCCCGGCGATCCTCTGG | Apramycin Cassette | This study |
| *flhD* pMDIAI Reverse | GAGATTCGCCTTACACGTTTACATCAATTTTTACAAATGTTGCATGACGGCAAGTGGACG | Apramycin Cassette | This study |
| *flhDC* pKSI-1 Forward | GATCTATCGAGGATCCTTTAGCTTTACTCTGTTTATCGCATTTCTGC | Plasmid Generation | This study |
| *flhDC* pKSI-1 Reverse | ATCGTAGTCTGTCGACGACATCATCCTTCCGCTGTTGACTATGAC | Plasmid Generation | This study |
| *flhDC* -278 A>T | GATTTTAGAAAATATGTGATGCAGAACACATATTTTAACGGAATACTTACGATAA | Site Directed Mutagenesis | This study |
| *flhDC* -278 A>T | TTATCGTAAGTATTCCGTTAAAATATGTGTTCTGCATCACATATTTTCTAAAATC | Site Directed Mutagenesis | This study |
| *dam* pWSK129 Forward | GATCTATCGAGGATCCTCCAGGCTGTGTCCTGCAATTGCCTGTGAGTGTC | Plasmid Generation | This study |
| *dam* pWSK129 Reverse | CGTAGCGTAAAAGCTTGAGAATTATTTTCTTGCAGGCGTTGCGACTCC | Plasmid Generation | This study |

1. Elhadad, D., Desai, P., Rahav, G., McClelland, M. and Gal-Mor, O. (2015) Flagellin Is Required for Host Cell Invasion and Normal Salmonella Pathogenicity Island 1 Expression by Salmonella enterica Serovar Paratyphi A. *Infect Immun*, **83**, 3355-3368.
